# CCL20/CCR6 signaling modulates disease severity during the establishment of *Staphylococcus aureus* osteomyelitis

**DOI:** 10.1128/mbio.01413-25

**Published:** 2025-08-25

**Authors:** Himanshu Meghwani, Javier Rangel-Moreno, Kyra M. Sandercock, Motoo Saito, Katya A. McDonald, Chloe M. Kraft, Robert Constantine, Sophia Lenigk, Adryiana Rodriguez, Stephen L. Kates, Jennifer H. Jonason, Edward M. Schwarz, Gowrishankar Muthukrishnan

**Affiliations:** 1 Department of Orthopedics, Center for Musculoskeletal Research, University of Rochester Medical Center 6923, Rochester, New York, USA; 2 Division of Allergy, Immunology and Rheumatology, Department of Medicine, University of Rochester Medical Center 6923, Rochester, New York, USA; 3 Department of Microbiology and Immunology, University of Rochester Medical Center 6923, Rochester, New York, USA; 4 Department of Orthopedics, Virginia Commonwealth University, Richmond, Virginia, USA; University of Colorado Anschutz Medical Campus, Aurora, Colorado, USA

**Keywords:** CCL20, CCR6, host immunity, T cells, *Staphylococcus aureus*, osteomyelitis

## Abstract

Chemokines are essential mediators of immune responses, and the CCL20/CCR6 chemokine signaling axis is known to be involved in inflammation, infectious diseases, and cancer progression. However, the role of the CCL20/CCR6 axis in host defense against *Staphylococcus aureus* osteomyelitis remains unknown. We hypothesized that the CCL20/CCR6 axis is critical for the recruitment and activation of immune cells against *S. aureus*, and the lack of CCL20 or its monogamous receptor CCR6 leads to exacerbation of *S. aureus* osteomyelitis. *In vitro* studies confirmed that osteoblasts and macrophages (M0 and M2 subtypes) secrete CCL20 following *S. aureus* exposure. Implant-associated osteomyelitis in C57BL/6, CCL20^−/−^, and CCR6^−/−^ mice revealed an early increase in planktonic bacterial growth on day 1 and increased bacterial loads in soft tissue and bone on day 14 post-infection in both CCL20^−/−^ and CCR6^−/−^ mice. Immunohistochemistry and flow cytometry revealed that CCL20^−/−^ and CCR6^−/−^ mice have impaired recruitment of T cells, especially CCR6^+^ T cells, to the site of infection. Interestingly, CCR6^−/−^ mice exhibited increases in osteoclast numbers, reactive bone formation, and reduced bone mineral density. In a clinical pilot study, we observed a fivefold increase in serum CCL20 levels (*P* < 0.05) in *S. aureus* osteomyelitis patients (*n* = 23) vs uninfected controls (*n* = 10). Remarkably, serum CCL20 levels immediately following septic death were 100-fold higher vs uninfected patients (*P* < 0.05). Collectively, these results highlight the critical role of CCL20/CCR6-mediated host immunity during the establishment of *S. aureus* osteomyelitis and the potential of CCL20 as a biomarker of osteomyelitis-induced sepsis.

**IMPORTANCE:**

*Staphylococcus aureus* is the most common pathogen in orthopedic infections, and hard-to-treat strains (methicillin-resistant *S. aureus*) cause >50% of these infections. Thus, there is an urgent need to develop immunotherapies to treat these life-threatening infections. The role of the CCL20/CCR6 chemokine signaling axis on *S. aureus* osteomyelitis is unknown. In our efforts to uncover its role, we reveal that osteoblasts and macrophages secrete CCL20 in response to infection, and mice lacking CCL20 or its monogamous receptor CCR6 are more susceptible to *S. aureus* osteomyelitis. Mechanistically, we observed that increased infection severity in the knockout mice is associated with decreased T cell recruitment and increased osteoclastogenesis at the bone infection site. Importantly, in a clinical pilot study, we observed that CCL20 can be a useful biomarker of osteomyelitis-induced septic death. Overall, our study highlights the crucial immunomodulatory role that the CCL20/CCR6 axis plays during osteomyelitis.

## INTRODUCTION

Implant-associated osteomyelitis is characterized as an infection associated with the presence of orthopedic implants, such as prosthetic joints, plates, or screws. Despite 50 years of significant advances in orthopedic surgical techniques, infection rates due to implant-associated osteomyelitis in elective surgery patients remain at 0.8%–2.9% (1–3), and developing solutions to this catastrophic outcome for patients and great costs to healthcare systems has been the focus of recent international consensus meetings (4–7). *Staphylococcus* species (*S. aureus and S. epidermidis*) account for most implant-associated infections, including 10,000–20,000 prosthetic joint infections (PJIs) per year, and 30%–42% of all fracture-related infections in the United States (8–10). Bacterial colonization of the implant surface leads to persistent infection and biofilm formation, which are hard to treat with antibiotics and often require surgical intervention (11). Unfortunately, even with optimized clinical management, 14%–25% of *S. aureus* osteomyelitis cases result in revision surgeries, which are associated with high recurrence rates and devastating outcomes such as amputation and sepsis (12). With the increasing incidence of methicillin-resistant *S. aureus* (MRSA) osteomyelitis and emerging strains with pan-drug resistance (13–15), there is an urgent need for novel therapies to supplement existing antibiotic therapies.  

The catastrophic outcomes of *S. aureus* osteomyelitis include osteolysis and septic death (16, 17), and the immune mechanisms associated with *S. aureus* osteomyelitis-induced sepsis are largely unknown. An early increase in plasma concentrations of cysteine–cysteine motif chemokine ligand 20 (CCL20) and its receptor C-C motif chemokine receptor 6 (CCR6) was reported in patients with sepsis; their levels correlated with disease severity, with the highest concentrations observed in patients admitted to the intensive care unit (ICU) due to severe sepsis (18). CCL20, also known as macrophage inflammatory protein-3 alpha (MIP-3α) or LARC, is a small protein belonging to the CC chemokine family (19). It is produced by various cell types, including epithelial cells, dendritic cells, and macrophages (19), in response to inflammatory stimuli. CCL20 exerts its effects by binding to its monogamous receptor, CCR6, which is expressed on the surface of various immune cells, including dendritic cells, Th17 cells, memory T cells (Tmem), regulatory T cells (Tregs), and B cells (20, 21). CCR6 is required for the directional migration of cells toward sites of CCL20 production (22). CCL20/CCR6 signaling through phosphorylation of Akt, mTOR, and STAT3 molecules has been implicated in regulating T cell differentiation, activation, and migration (23). Th17 cells, which play a critical role in host defense against extracellular pathogens and autoimmune diseases, are attracted to CCL20-rich environments via CCR6 (23). This interaction has been implicated in various diseases, including cancer, autoimmune disorders, and inflammatory conditions (24, 25). CCL20, IL-8, and IL-6 are among the proteins increased in PJI vs non-infectious arthroplasty failure sonicated fluid samples (26).

Besides its role in chemotaxis, the CCL20/CCR6 axis maintains bone homeostasis and enhances osteoblast-mediated osteoclastogenesis via IL-6 production, suggesting that CCL20 may contribute to bone loss in rheumatoid arthritis (27). CCR6 also influences bone metabolism. For example, CCR6 deletion reduces osteoblast differentiation via downregulation of the transcription factor Osterix and indirectly promotes osteoclast production by increasing RANKL (28). Another study confirmed that CCL20/CCR6 signaling regulates bone mass accrual by modulating the survival and maturation of osteoblasts and through the recruitment of osteoblast-supporting cells, T cells, and macrophages, which facilitates osteoblast differentiation (29). Currently, the role of the CCL20/CCR6 axis during *S. aureus*-osteomyelitis is unknown. Since the CCL20/CCR6 axis regulates bone turnover and participates in the efficient induction of protective immunity, we hypothesized that this axis is essential for ameliorating the severity of *S. aureus* osteomyelitis.

## RESULTS

### Osteoblasts and macrophages secrete CCL20 in response to *S. aureus*

**Fig 1:**
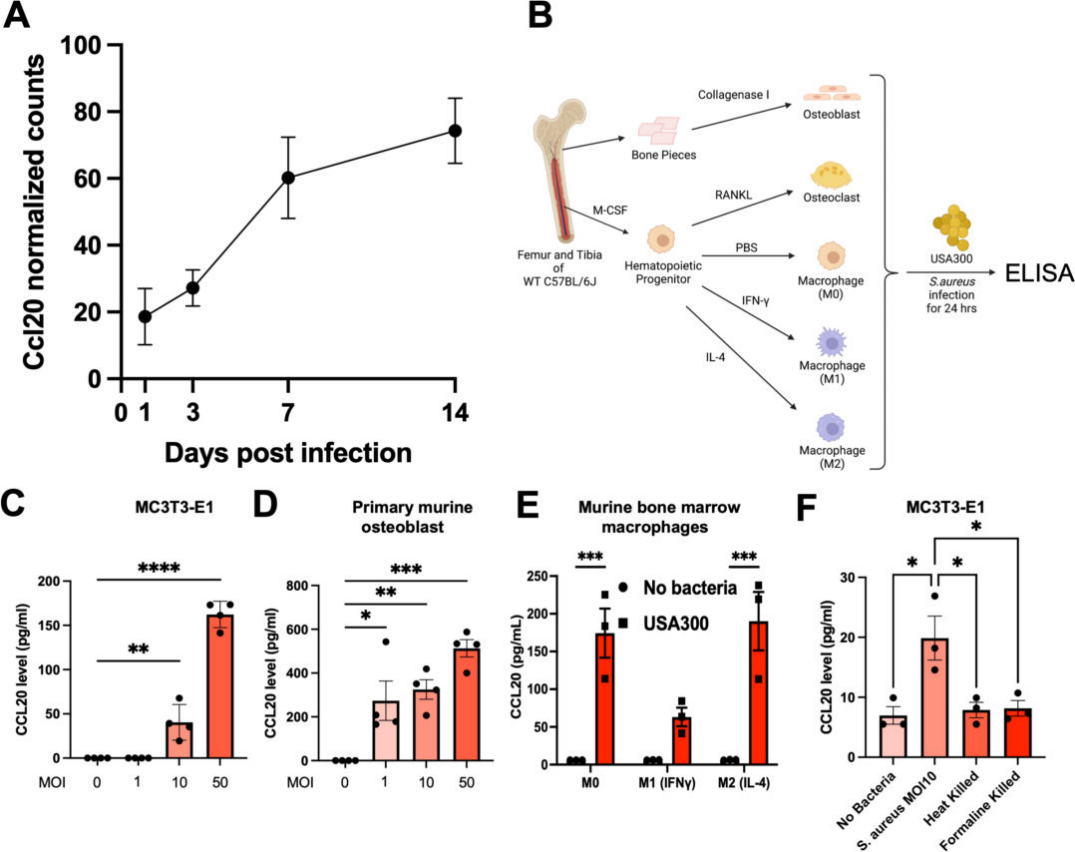
CCL20 is produced in response to *S. aureus* infection. (**A**) RNA isolation from MRSA-infected tibiae showed a progressive increase in normalized counts of CCL20 over time (*n* = 3 at each time point, *P* < 0.05). (**B**) Experimental design to examine CCL20 production *in vitro* in various *S. aureus*-infected bone cells. (**C**) Murine calvarial MC3T3-E1 osteoblasts were exposed to *S. aureus* USA300 (multiplicity of infection [MOI] = 1, 10, and 50), and CCL20 was measured in culture supernatants by enzyme-linked immunosorbent assay (ELISA). (**D**) Primary bone marrow-derived osteoblasts were challenged with *S. aureus* USA300 (MOI = 1, 10, and 50) for 24 h, and CCL20 was measured by ELISA in culture supernatants. (**E**) Primary murine bone marrow-derived macrophages stimulated with phosphate buffered saline (PBS), IFN-γ (50 ng/mL), or IL-4 (20 ng/mL) to generate M0, M1, and M2 macrophages, respectively, were exposed to *S. aureus* USA300 for 24 h (MOI  =  10). CCL20 was measured in culture supernatants by ELISA. (**F**) The MC3T3 –E1 osteoblast was challenged with an MOI of 10 live bacteria, heat-killed, and formalin-killed bacteria for 24 h, and CCL20 levels were measured in the supernatant using ELISA (*n* = 3 biological replicates, and the test was performed in duplicates). The data from each experiment represent the mean  ±  SEM for the group. Statistical significance was calculated with one-way analysis of variance (ANOVA) (*P  < 0.05, **P < 0.01, ***P <   0.001, and ****P  <  0.0001).

There is evidence that the CCL20/CCR6 axis is important for the induction of protective immunity against various pathogens in various organs (30). However, the kinetics of CCL20 expression and the identity of the cellular sources of CCL20 during murine osteomyelitis are unknown. Thus, we examined the mRNA expression of *Ccl20* in the bone at different times post-transtibial *S. aureus* infection in mice. We explored our previously published RNA sequencing data (GSE168896) and found a steady and progressive increase in normalized counts of *Ccl20* transcripts 1, 3, 7, and 14 days post *S. aureus* infection (Fig. 1A). Quantitation of CCL20 protein in the serum of C57BL/6 and CCL20^−/−^ mice on day 14 post-infection confirmed the efficient translation of *Ccl20* mRNA in C57BL/6 mice and its compromised expression in CCL20^−/−^ mice (Fig. S1).

Next, we sought to identify the cellular sources of CCL20 during *S. aureus*-induced osteomyelitis. Murine calvarial MC3T3-E1 osteoblasts, primary bone marrow-derived osteoblasts, osteoclasts (M-CSF+RANKL), and macrophages (M0: PBS, M1: IFNγ, and M2: IL4) in culture (Fig. 1B). Next, we challenged these cell cultures with *S. aureus* USA300 (MOI = 1, 10, and 50) for 24 h, and CCL20 was measured in the supernatant by ELISA. Interestingly, both murine calvarial MC3T3-E1 osteoblasts and primary bone marrow-derived osteoblasts expressed CCL20 in response to *S. aureus* in a concentration-dependent manner (Fig. 1C and D). Similarly, M0 and M2 macrophages secreted CCL20 *in vitro* after *S. aureus* infection (MOI = 10; Fig. 1E). We further confirmed the impact of live vs dead bacteria on CCL20 production by osteoblastic cells. We challenged MC3T3-E1 cells with live, heat-killed (75°C for 30 min), or formalin-killed/fixed bacteria (10% for 30 min; 10 MOI). Interestingly, significant CCL20 was observed only when challenged with live bacteria (Fig. 1F). To our knowledge, this is the first demonstration of CCL20 production by osteoblasts in response to live *S. aureus*, highlighting the relevant contribution of osteoblast-derived CCL20 in the efficient recruitment of CCR6^+^ immune cells to the site of bone infection. In addition, transtibial murine osteomyelitis studies also confirmed a higher number of CCL20-producing cells in the bone sections around abscesses in *S. aureus*-infected WT and CCR6^−/−^ mice (Fig. S2).

### CCL20/CCR6 axis is critical for controlling *S. aureus* growth during osteomyelitis

**Fig 2:**
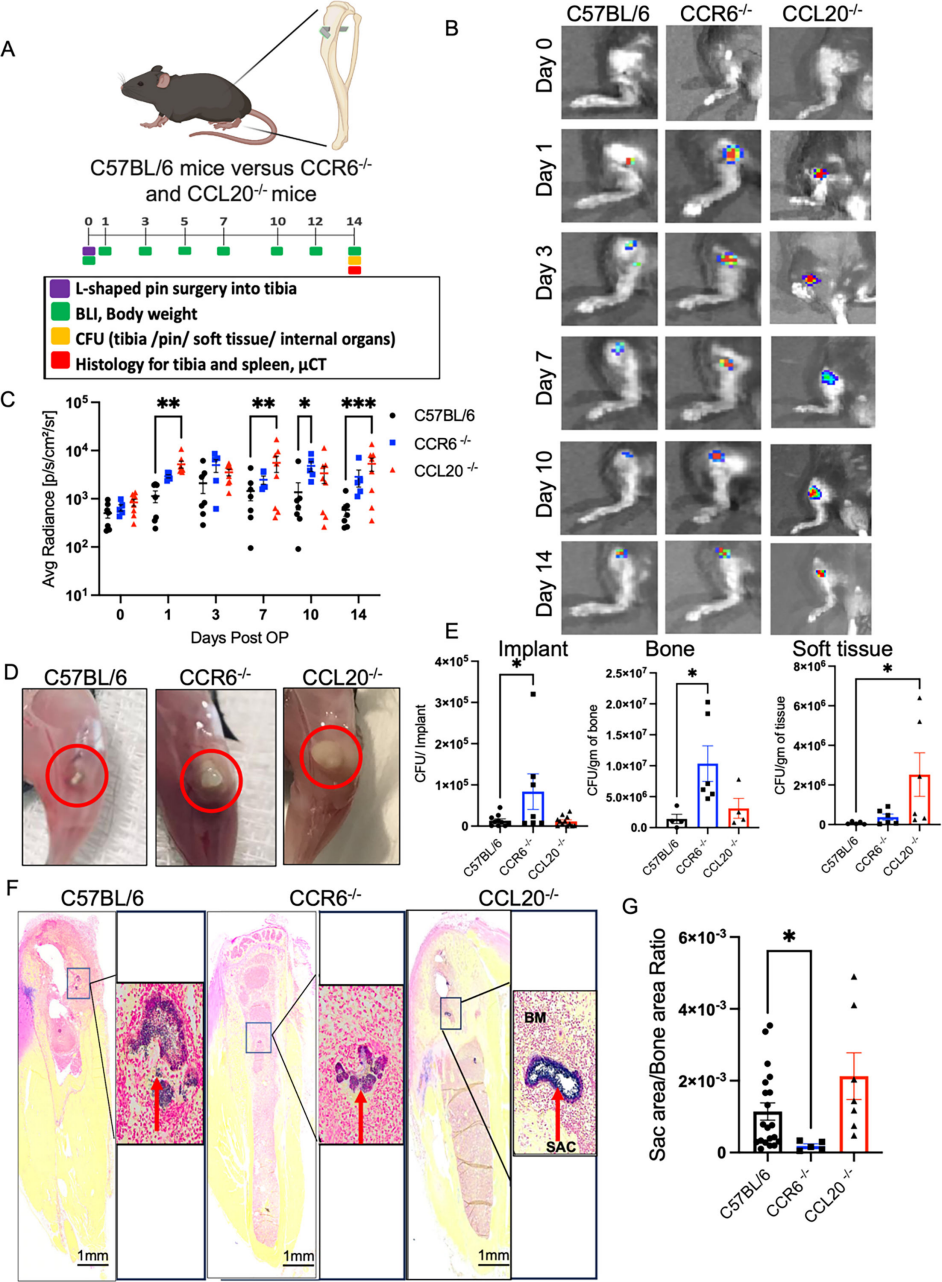
CCL20/CCR6 axis is essential to modulating infection severity at the surgical site during *S. aureus* implant-associated osteomyelitis. (**A**) Schematic illustration of the experimental design in which 8−10 weeks old male and female C57BL/6, CCR6^−/−^, and CCL20^−/−^ mice were challenged with an MRSA (2 × 10^5^ CFU of USA300 LAC::lux) contaminated transtibial implant. Time points for the various outcome measures are also indicated. (**B**) Longitudinal BLI images and (**C**) quantitative measurements showing average radiance represent the mean ± SEM (*n* = 5–8 mice/group, two-way ANOVA, *P < 0.05, **P < 0.01, ***P < 0.001, and ****P < 0.0001). (**D**) Representative images of infected tibiae obtained on day 14 post-infection illustrate the size differences of the abscesses in C57BL/6, CCR6^−/−^, and CCL20^−/−^ mice. Mice were euthanized on day 14 after infection, and tibiae were harvested for CFU quantitation. (**E**) At day 14 post-infection, terminal *ex vivo* CFU quantification of *S. aureus* was performed on the implant, tibia, and soft tissue surrounding the tibia. Data are presented as mean ± SEM (*n* = 4–10 mice/group, one-way ANOVA, *P < 0.05). (**F and G**) Tibial sections were stained with Brown and Brenn gram staining, and the ratio between the SAC area and bone area was calculated for each experimental group. Data are presented as mean ± SEM (*n* = 6–8 mice/group and 2–3 levels/tibia, one-way ANOVA, *P < 0.05).

We next used C57BL/6, CCL20^−/−^, and CCR6^−/−^ mice to demonstrate the functional relevance of the CCL20/CCR6 axis in the control of the bacterial burden in the mouse model of osteomyelitis. Experimental timeline and outcomes are shown in Fig. 2A. Body weight analyses as a measure of morbidity revealed comparable declines in mass across all experimental groups at early stages, which appear to gradually improve over the 14 days post-infection in C57BL/6 and CCR6^−/−^ mice, though not significantly (Fig. S3). However, *in vivo*, *S. aureus* growth, assessed by bioluminescent intensity (BLI) on days 1, 3, 7, 10, and 14 after infection, showed an early increase in planktonic bacterial burden in CCL20^−/−^ and CCR6^−/−^ mice, compared to C57BL/6 mice (Fig. 2B and C). Interestingly, mice lacking CCL20 or CCR6 displayed larger draining abscesses than C57BL/6 animals (Fig. 2D). *Ex vivo* CFU analyses confirmed that the bacterial load was significantly higher in the bone (*P* < 0.05) and the implant (*P* < 0.05) from CCR6^−/−^ mice, while bacterial burden was significantly higher in soft tissue (*P* < 0.05) of CCL20^−/−^ mice, compared to C57BL/6 controls (Fig. 2E).

Staphylococcal abscess communities (SAC) formation depends on the bacteria’s capacity to evade the host immune system and the ability of the bone tissue to prevent bacterial dissemination to other organs (31). Therefore, we utilized histomorphometry of bone sections to estimate the area occupied by SAC in the infected bone. Unexpectedly, we noticed that infected bones of CCR6^−/−^ mice had a smaller SAC area compared to CCL20^−/−^ and C57BL/6 mice at day 14 post-infection (Fig. 2F through G; *P* < 0.05). Of note, we did not detect any BLI signal or CFU in the internal organs, which suggests that smaller SAC areas in CCR6^−/−^ mice are not associated with systemic bacterial dissemination.

The bone homogenates were also used to measure the levels of various cytokines during infection. Interestingly, IL-17A concentration was significantly higher in both CCL20^−/−^ and CCR6^−/−^ mice, compared to C57BL/6 mice (*P* < 0.05), which indicates IL-17-producing cells are still able to accumulate at the site of infection. Other cytokines showing significant differences in both the mouse strains were IL-33 and TNFβ. Meanwhile, IL-28B and IL-31 showed a higher level only in the CCL20^−/−^ mice (Table 1).

**TABLE 1:** Cytokine levels in bone homogenates 14 days post-infection^*a*^

Cytokine	C57BL/6 (pg/mL)	CCR6^−/−^ (pg/mL)	CCL20^−/−^ (pg/mL)
IL-17A	4.4 ± 2.3	17.6 ± 8 **	94 ± 31.7 ***
IL-17E	2.6 ± 0	97.8 ± 30.2	258.3 ± 133.5
IL-17F	1.1 ± 0.8	3.1 ± 0.6	5 ± 2
IL-21	21.9 ± 14	48.8 ± 11.3	46.1 ± 5.3
IL-22	2.9 ± 1.8	1.4 ± 0.3	1.9 ± 0.7
IL-23	139.5 ± 34.8	453.3 ± 106.1	407.5 ± 170.6
IL-27	47.9 ± 22.3	312 ± 38.3	353 ± 161.6
IL-28B	52.5 ± 42.7	271.6 ± 39.6	327.2 ± 94.9 *
IL-31	4.7 ± 0.2	23.5 ± 6.6	73.2 ± 26.1 *
IL-33	601.8 ± 334.6	2130 ± 431.4 *	1583.5 ± 486.1 *
TNFβ	237.4 ± 125.2	503.6 ± 23.7 ****	435 ± 82.4 ****

^*a*^ Cytokine concentrations (in pg/mL) were measured in bone homogenates collected from three experimental groups 14 days after infection. Data are expressed as mean ± SEM (*n* = 5 per group). Statistical significance was determined using one-way ANOVA (*P < 0.05; **P < 0.01; ***P < 0.001; ****P < 0.0001).

### The CCL20/CCR6 ligand-receptor pair is essential for the recruitment and positioning of CCR6^+^ T cells at the site of infection

**Fig 3:**
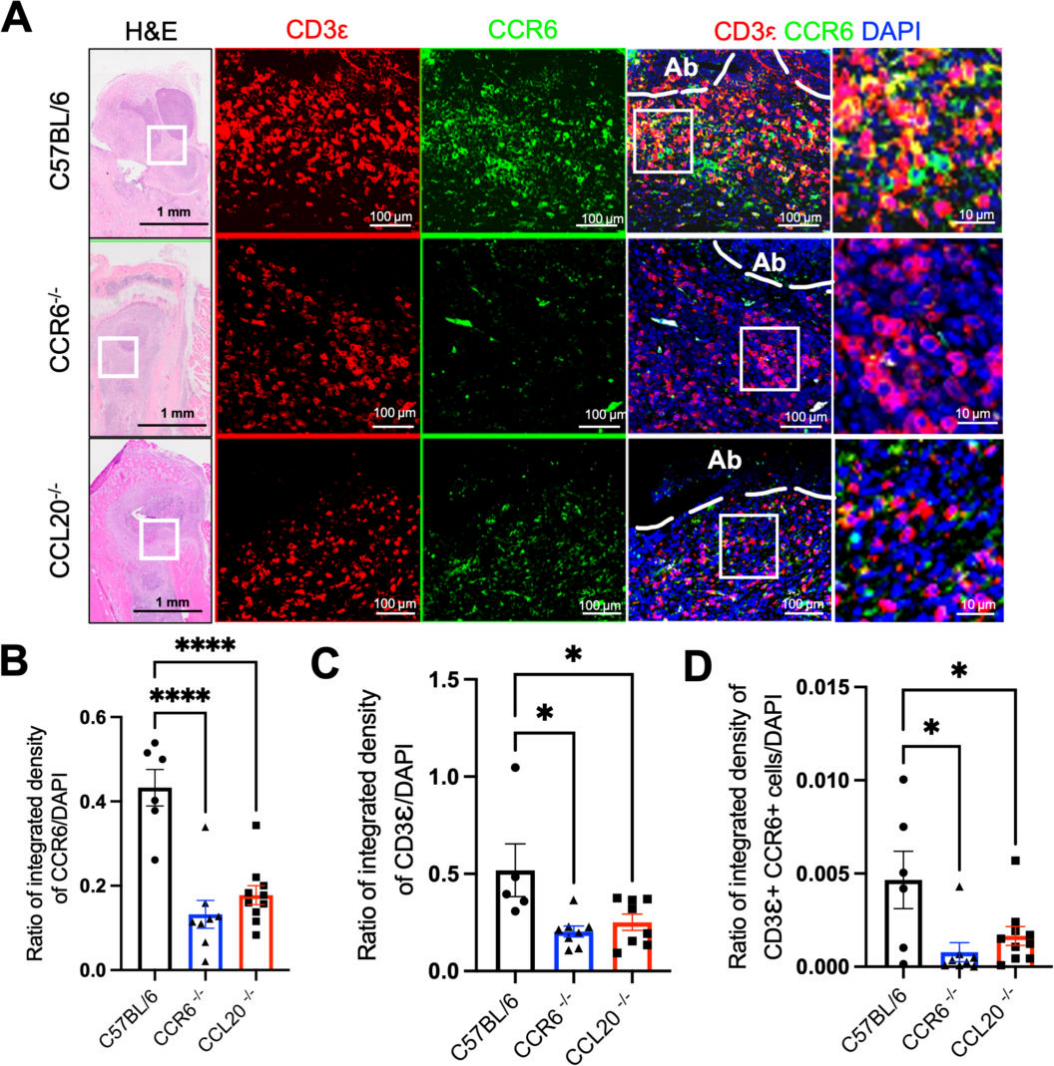
CCL20 and CCR6 are required for optimal accumulation of T cells at the site of infection in mice with implant-associated *S. aureus* osteomyelitis. (**A**) Tibiae from C57BL/6, CCR6^−/−^, and CCL20^−/−^ mice challenged with an MRSA-contaminated implant were harvested on day 14 post-infection and stained by immunofluorescence. Representative hematoxylin and eosin (H&E) images of tibial sections containing abscesses adjacent to the pin were stained with antibodies specific for T cells (CD3ε in red) and CCR6 (green). Nuclei were labeled with DAPI (blue). Scale bar = 100  µm. Histomorphometric analysis was performed to calculate the ratio of raw integrated density between (**B**) CCR6 and DAPI, (**C**) CD3ε and DAPI, and (**D**) CCR6^+^CD3ε^+^ T cells. Data represent the mean ± SEM for the groups (*n* = 3–4 mice/group and 2–3 levels/tibia). One-way ANOVA was utilized to calculate statistical significance (*P < 0.05 and ****P < 0.0001).

**Fig 4:**
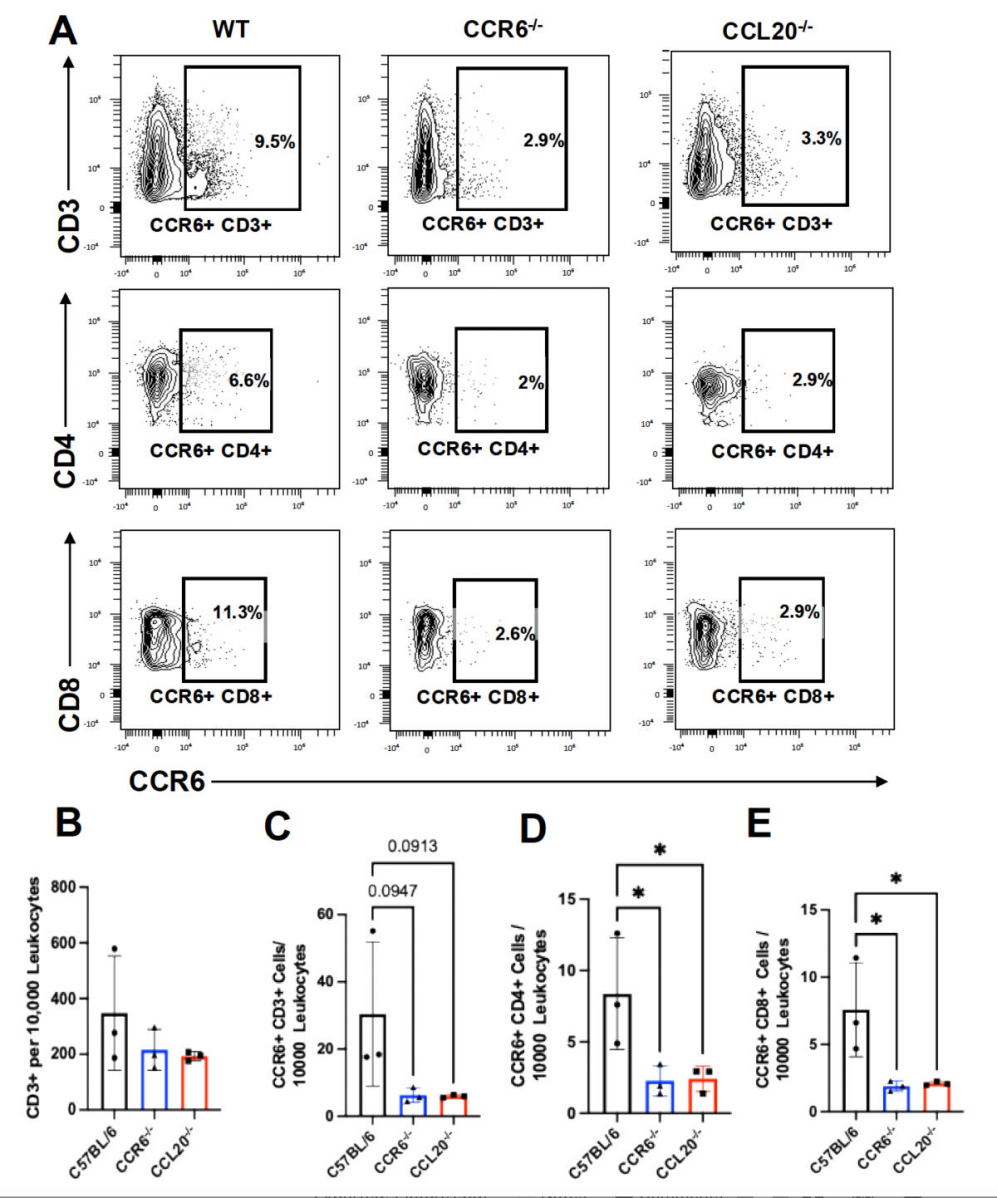
Flow cytometry confirms the recruitment of T cells and CCR6^+^ T cells at the site of infection, which requires the CCL20-CCR6 axis during implant-associated *S. aureus* osteomyelitis. (A) A multichromatic spectral flow cytometry assay was developed, optimized, and performed on C57Bl/6, CCL20^−/−^, and CCR6^−/−^ mice’s tibial BMCs. CD45^+^/CD3^+^/T cells and their subpopulations (CCR6^+^ CD4^+^ T cells) were analyzed. The frequency of (B) CD3^+^ T cells and (C) CD3+/CCR6^+^ T cells in CCL20^−/−^ and CCR6^−/−^ mice shows a trend compared to C57BL/6 mice. Moreover, T cell subpopulations, including (D) CCR6^+^ CD4^+^ T cells and (E) CCR6^+^ CD8^+^ T cells, are significantly low in CCL20^−/−^ and CCR6^−/−^ BMCs as compared to controls 14 days after MRSA infection (*n* = 3 mice/group). One-way ANOVA was utilized for calculating statistical significance (*P < 0.05 and **P < 0.01).

CCR6 is expressed by several immune cells, including T cells and macrophages (32, 33), and mediates chemotaxis toward the CCL20 gradient at the infection site. Thus, we next focused on examining the spatial location of CCR6^+^ T cells and macrophages near the abscesses. Immunofluorescence and histomorphometry were performed on infected tibial sections, which showed a reduction in T cell recruitment at the site of infection in CCR6^−/−^ and CCL20^−/−^ mice compared to the C57BL/6 controls (Fig. 3A and C). We identified the ratio of CCR6^+^ cells/DAPI, CCR6^+^CD3ε^+^ T cells/DAPI intensity (Fig. 3B through D) near the abscesses to be significantly reduced in CCR6^−/−^ and CCL20^−/−^ mice compared to C57BL/6 mice. Of note, we confirmed that the DAPI area per field analyzed is consistent across the three genotypes (Fig. S4), suggesting that the observed reduction is not influenced by the cellular density at the site of the infection. Next, to confirm our immunohistochemistry (IHC) findings, we performed multicolor spectral flow cytometry on bone marrow cells (BMCs) isolated 14 days post-infection. Total CD3^+^ T cells and CCR6^+^CD3^+^ T cells were reduced in both CCR6^−/−^ and CCL20^−/−^ mice, compared to C57BL/6 mice (Fig. 4B and C). Even the number of CCR6^+^CD4^+^ T cells (Fig. 4D) and CCR6^+^CD8^+^ T cells (Fig. 4E) was significantly reduced in the whole bone marrow sample.

Immunofluorescence also revealed a significant reduction in the density of F4/80^+^ macrophages near the SAC regions in the infected CCR6^−/−^ and CCL20^−/−^ mice compared to the C57BL/6 controls. The intensity of CCR6^+^F4/80^+^ macrophages was reduced at the infection site (Fig. S5A through C). However, flow cytometry analyses showed that the number of macrophages was comparable among the groups (Fig. S6A and B).

Collectively, our results indicate a global impairment in the recruitment and strategic positioning of CCR6^+^ T cells into the *S. aureus*-infected bones.

### CCR6^−/−^ mice display altered bone remodeling during infection

**Fig 5:**
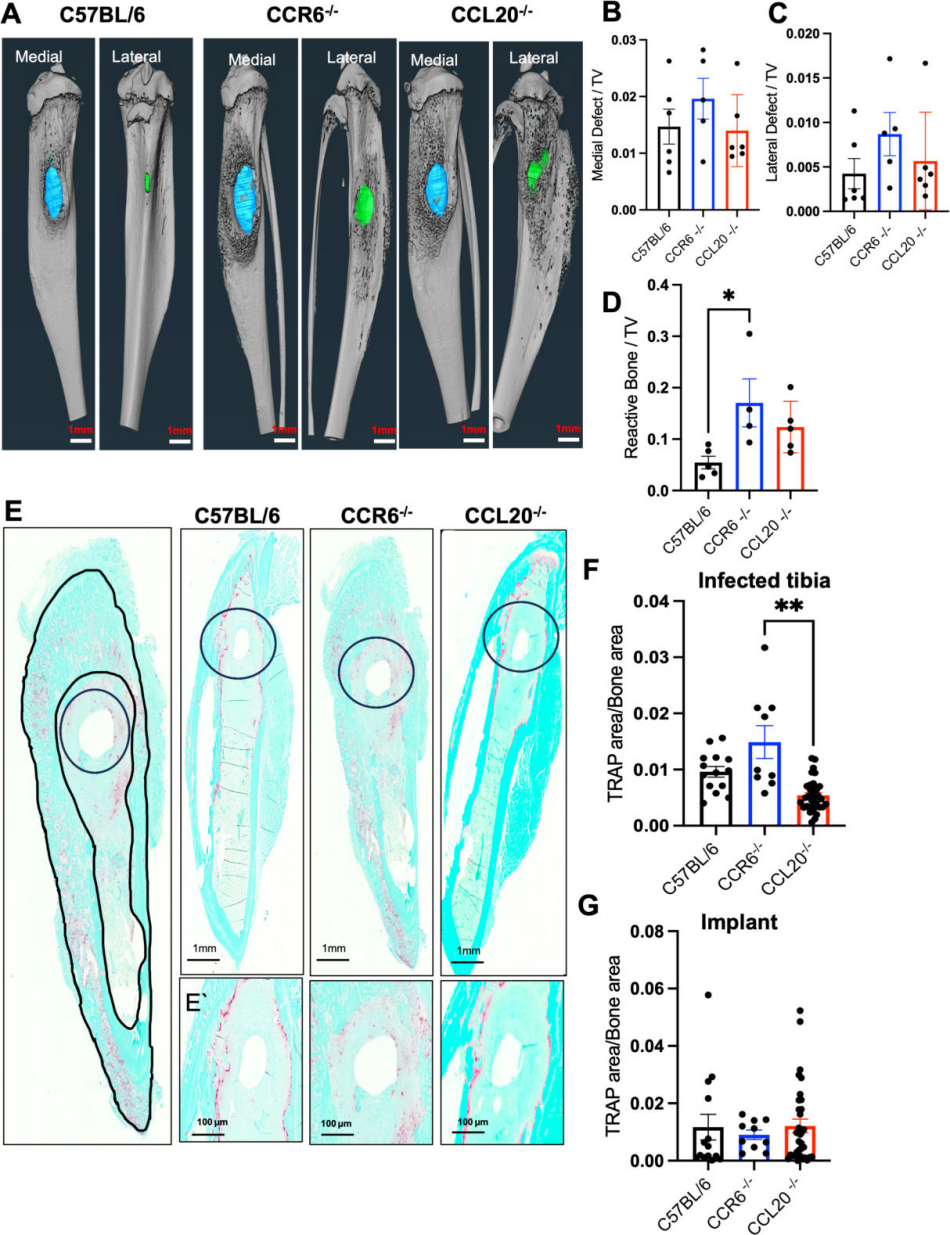
CCR6^−/−^ and CCL20^−/−^ mice display altered bone remodeling. Bone osteoclastic and osteoblastic activity was measured by using μCT, TRAP stain, and DEXA scan. (**A**) Bone μCT imaging was performed to show representative 3D renderings of extensive peri-implant reactive bone formation and bone defect (lateral and medial) at 14 days post-infection. Quantitative measurements of (**B**) medial and (**C**) lateral volume of bone loss and (**D**) reactive bone formation are shown. Data for each tibia represent the mean ± SEM for the group (*n* = 4–6 mice/group, *P < 0.05, ANOVA). (**E**) Tibiae from the mice were decalcified and embedded in paraffin. Bone sections were stained with TRAP. Representative images are shown with a magnification of 20× and scale bars = 1 mm. The highlighted area in the tibia shown in the representative image from the CCR6^−/−^ group image (used for illustrative purposes only) defines the whole bone area and the implant site used to calculate the TRAP-positive area. The ratio of TRAP-stained areas to bone area was calculated for (F) whole bone and (**G**) the implant site, depicting an increased osteoclastic activity in CCR6^−/−^ mice. Data for each tibia represent the mean ± SEM for the groups (*n* = 4 mice/group and 2–4 levels/tibia). One-way ANOVA was utilized to calculate statistical significance (*P < 0.05, **P < 0.01, ***P < 0.001, and ****P  < 0.0001).

Next, we examined the relationship between bone morphology, *S. aureus* infection, and the CCL20/CCR6 axis using micro-computed tomography (μCT) imaging (Fig. 5A). We did not observe any difference in the volume of bone loss on either medial or lateral bone sides (Fig. 5A through C). However, CCR6^−/−^ mice showed increased reactive bone formation at the pin insertion site (Fig. 5A through D) vs C57BL/6 and CCL20^−/−^ mice. Increased reactive bone formation could be attributed to the change in osteoclast or osteoblast activity in the bone microenvironment of the CCR6^−/−^ mice. To confirm this, we stained the bone sections for tartrate-resistance acid phosphatase (TRAP) and measured the ratio of TRAP-positive area to bone area (Fig. 5E and E’). Interestingly, CCR6^−/−^ mice had overall increased osteoclastic area compared to the CCL20^−/−^ and C57BL/6 mice at day 14 after infection (Fig. 5F). Furthermore, this difference was not observed around the implant site (Fig. 5G). Enhanced reactive bone formation was further confirmed using dual-energy X-ray absorptiometry (DEXA), which showed a decreasing bone mineral density (BMD) trend in C57BL/6 mice and an increasing trend in the CCR6^−/−^ mice 14 days post-infection (Fig. S7).

### Serum CCL20 levels were associated with adverse outcomes in patients with *S. aureus* osteomyelitis

**Fig 6:**
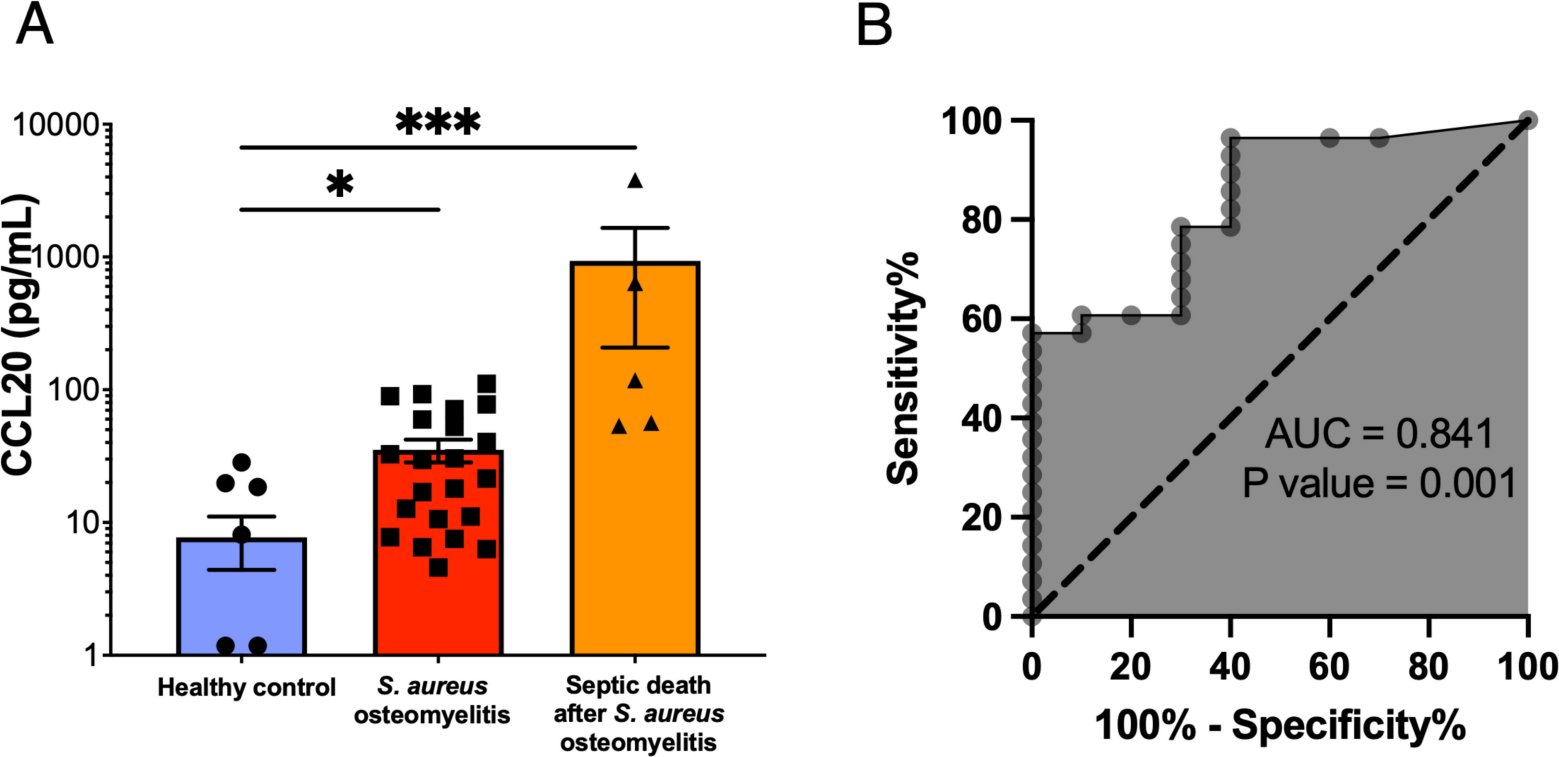
Serum CCL20 is a non-invasive biomarker of *S. aureus* infection severity during osteomyelitis. (**A**) Sera were collected from healthy individuals (*n* = 10), orthopedic patients with culture-confirmed *S. aureus* bone infections (*n* = 23), and patients who died from septic *S. aureus* osteomyelitis (*n* = 5). CCL20 was quantitated in sera by using a Luminex assay. Data for each sample represent the mean ± SEM for each experimental group. (**B**) Luminex data were utilized to generate a receiver operating characteristic curve, and the areas under the curve for the controls and infected patients are presented. CCL20 concentration is highly predictive of *S. aureus* osteomyelitis. The dashed line represents a nondiscriminatory test with equal sensitivity and specificity (one-way ANOVA; *, *P* < 0.05; ***, *P* < 0.001).

To investigate the clinical relevance of the CCL20/CCR6 axis in human osteomyelitis, we measured CCL20 in the sera of orthopedic patients with culture-confirmed *S. aureus* osteomyelitis, patients who died from osteomyelitis-induced sepsis, and patients who underwent total joint arthroplasties but did not develop post-operative infections (control patients). In the control patient group, which consisted of six females and four males, the average age was 61 years (±2.5), and body mass index (BMI) was 32.67 (±4.9). The *S. aureus* osteomyelitis group consisted of 8 females and 15 males with an average age of 61.4 years (±17.6) and a BMI of 31.03 (±7.9). The cohort of septic death patients consisted of three females and two males with an average age of 60.2 years (±19.4) and a BMI of 22.96 (±2.7). Serum CCL20 levels were fivefold higher in infected patients than in uninfected controls (Fig. 6A). Remarkably, CCL20 levels immediately following septic death were ~100-fold higher than in uninfected controls (Fig. 6A). These findings suggest that serum CCL20 levels could be a useful predictor of disease progression, especially *S. aureus* osteomyelitis-induced septic death. Additionally, using receiver operating characteristic curve analysis, we examined whether CCL20 serum levels can be utilized as a prognostic biomarker for identifying *S. aureus* osteomyelitis. Indeed, our analyses demonstrated that CCL20 serum levels could predict *S. aureus* bone infections with high sensitivity and specificity, summarized by an area under the curve of 0.84 (Fig. 6B).

## DISCUSSION

The CCL20/CCR6 axis is known to be involved in autoimmune diseases, cancer, and inflammatory diseases, including inflammatory bowel disease, multiple sclerosis, rheumatoid arthritis, and psoriasis (20, 24, 25). In the current study, we demonstrated that the CCL20/CCR6 axis is essential in the induction of protective immunity to *S. aureus* and in modulating bone pathogenesis during osteomyelitis. The interaction between the immune system and bone in osteomyelitis involves elaborating cytokines that alter the phenotypic expression of bone cells. Both macrophages and osteoclasts release cytokines and chemokines in response to infection, contributing to osteolysis and tissue damage. They also contribute to the increased recruitment of immune cells to the site of inflammation and aid in clearing the bacteria in mice (34). Here, we observed that *S. aureus* infection induces the secretion of CCL20 in macrophages, and subtypes M0 and M2 can secrete significantly higher amounts of CCL20 in the supernatant after being challenged with *S. aureus* compared to the M1 subtype. Other studies have shown that both M1 and M2 macrophages can secrete CCL20 after interaction with melanoma cells and tumor-derived factors (35), and lipopolysaccharides (LPS) can stimulate the production of CCL20 from M2 more than M1 macrophages (36). Notably, we discovered that osteoblasts secrete CCL20 when challenged with *S. aureus*. In the bone, it was shown that CCL20 and CCR6 expression increased only after the differentiation of bone marrow stromal cells toward the osteoblast lineage (29). Loss of either *Ccl20* or *Ccr6* results in reduced osteoblast numbers and decreased trabecular bone mass. CCL20/CCR6 signaling supports osteoblast differentiation, survival, and the recruitment of osteoblast-supporting cells like macrophages and T cells (29).

We observed that both CCR6^−/−^ and CCL20^−/−^ showed a higher disease severity than C57BL/6 mice. However, differences between the knockout strains suggest distinct chemotaxis-independent functions. In general, CCL20^−/−^ demonstrated better infection control than CCR6^−/−^ mice. Several reasons could be at play here. CCL20 has been reported to exhibit direct antimicrobial activity, primarily against gram-negative bacteria at low concentrations. It exhibits poor antimicrobial activity against *Candida albicans*, Group B *Streptococcus*, *S. epidermidis*, clinical strains of methicillin-sensitive *S. aureus*, and *Enterococcus* species (37). In our *in vitro* antibacterial assay, CCL20 directly inhibited bacterial growth only at relatively high concentrations (MIC_50_ 6.5–3.125 µg/mL), which are unlikely to be achieved under physiological or pathological conditions (Fig. S8). In sharp contrast, CCR6 contributes to host defense and direct killing of bacteria via the beta defensin pathway (19). The more severe phenotype, such as reactive bone formation, bioluminescence, and CFU in soft tissue in CCR6^−/−^ mice, may be attributed to the loss of this important bactericidal function (19, 37). Interestingly, we also observed elevated levels of IL-28B and IL-31 in CCL20^−/−^ mice, which could contribute to enhanced bacterial clearance through CCR6-independent pathways. IL-28B is known to inhibit *S. aureus* colonization through STAT1 signaling and ROS production in keratinocytes, while IL-31 is known to modulate macrophage function and control inflammation, potentially promoting host defense (38, 39). Taken together, these findings underscore the functional divergence between CCL20 and CCR6, an important avenue for future investigation.

The chemotactic potential of CCL20/CCR6 signaling axis is essential for orchestrating immunity against bacterial infections such as periodontitis (40), *Helicobacter pylori* gastritis, maintenance of adaptive immunity against bacteria in the gut (41), *Pseudomonas aeruginosa* peritonitis infections (42), and pneumococcal meningitis (43). It has been shown that disruption of this axis by using antibodies or antagonists prevents the migration of CCR6-expressing immune cells at the site of inflammation and reduces the severity of the disease (25). We demonstrated that genetic disruption of the CCL20/CCR6 axis impairs migration of T cells and may be macrophages to the site of infection during implant-associated *S. aureus* osteomyelitis. The recruitment and spatial organization of CCR6^+^ T cells and CCR6^+^ macrophages depended on the presence of CCL20 ligand. This defective recruitment and altered localization of key immune cells likely compromise bacterial clearance. Indeed, planktonic bacterial load was significantly higher in the CCL20^−/−^ and CCR6^−/−^ mice than in C57BL/6 mice.

A confounding observation in our IHC studies is the residual expression of CCL20 protein in the CCL20^−/−^ mice. The CCL20^−/−^ mouse strain (B6[C]-*Ccl20^tm1b(EUCOMM)Hmgu^*/KmskJ), obtained from JAX Laboratories, was generated through deletion of exon 2, which encodes amino acids 27–65. We verified this deletion by genotyping, following the recommended protocol (29). However, the antibody used to detect CCL20 was raised against a synthetic peptide spanning amino acids 31–97, which resulted in residual staining in the CCL20^–/–^ mice. This raised the possibility that these mice may express a mutant or truncated form of CCL20, which is likely non-functional. To investigate further, we infected wild-type C57BL/6, heterozygous, and homozygous CCL20^−/−^ mice with *S. aureus* and collected serum 14 days post-infection. ELISA confirmed low but detectable levels of CCL20 in homozygous knockout mice. Our results indicate that the CCL20^−/−^ strain may produce a truncated protein still recognized by the antibody, but at substantially reduced levels. While likely non-functional, this residual expression may influence disease progression by compromising bacterial clearance and likely contributing to increased disease severity.

Because CCR6 is commonly expressed on Th17 cells, we hypothesize that their recruitment would be compromised in CCL20^−/−^ and CCR6^−/−^ mice during osteomyelitis. Th17 cells induce the production of chemokines (IL-17) responsible for attracting neutrophils to the site of infection (44). IL-17 is also important in the induction of antimicrobial peptides (defensins and CRAMP) required to control *S. aureus* growth (45). Alternatively, it is likely that by affecting the recruitment of CCR6^+^ Th17 cells, the production of IL-17 and IL-17-induced antimicrobial peptides can be reduced in the infected bones, compromising the control of *S. aureus* infection. We detected cells with a neutrophil-like morphology, which were positive for CCL20 in C57BL/6 infected mice and not in knockout mice (data not shown). Thus, it is possible that neutrophils might attract CCR6^+^ Th17 to the site of *S. aureus* infection via CCL20 production (46) and accelerate bacterial clearance. CCR6^−/−^ mice exhibit a more severe phenotype than the CCL20^−/−^ mice, as shown by increased TRAP-stained area, increased reactive bone formation, and increased abscess formation. This difference may be attributed to the fact that CCL20 acts monogamously through the CCR6 receptor, while CCR6 alternatively gets activated by antibacterial peptides like beta-defensins (19). Moreover, we observed more CCR6^+^ T cells in the CCL20^−/−^ mice bone niche than in CCR6^−/−^ mice, suggesting compensatory or multiple chemokines playing a role in T cell recruitment to the bone niche. Our flow cytometry confirmed the impaired recruitment of total T cells and CCR6^+^ T cells to the site of infection in the tibia. Interestingly, macrophage numbers were comparable between the experimental groups. Perhaps the spatial positioning of macrophages in the infected tibia may be influenced by CCR6 expression on their surface and the establishment of a local CCL20 gradient, facilitating efficient bacterial clearance. Nonetheless, the spatial distribution of macrophages and other immune cells needs to be examined in greater detail using advanced techniques such as spatial transcriptomics and high spatial resolution matrix-assisted laser desorption/ionization (MALDI) imaging mass spectrometry (47).

The importance of the CCL20/CCR6 axis in maintaining bone mass accrual has been studied previously (29). Using knockout mice, Doucet et al. showed that the loss of CCL20/CCR6 signaling affected osteoblast maturation and survival and the recruitment of osteoblast-supporting cells, ultimately resulting in reduced trabecular bone mass (29). The authors also demonstrated that the signaling axis influenced osteoclastic activity but not their numbers (29). Other studies using noninfectious inflammation-related disease models (e.g., rheumatoid arthritis and cancer) showed that MIP family members, including CCL20, mediate pathological bone loss by promoting osteoclast formation (48, 49). We showed through TRAP staining that CCR6^−/−^ mice had overall increased osteoclastic area compared to the CCL20^−/−^ and C57BL/6 mice at day 14 after infection. TRAP is a recognized osteoclast marker that is also expressed in osteoblasts and osteocytes (50), and its role in bone metabolism appears to be context dependent. For instance, although elevated serum TRAP levels generally correlated with reduced BMD in metabolic bone diseases (51), transgenic overexpression of TRAP has been associated with both increased cortical BMD via elevated osteoblast activation (52) and decreased trabecular bone formation (53). We observed a lower BMD at baseline in CCR6^−/−^ mice compared to the C57BL/6 and CCL20^−/−^ mice. However, 14 days post *S. aureus* infection, we observed a reduction in BMD in C57BL/6 mice and increased BMD, reactive bone formation, and overall osteoclastic activity in CCR6^−/−^ mice. These results suggest that in the setting of implant-associated osteomyelitis, the complexity of osteoblastic-osteoclastic activity is further heightened due to the presence of *S. aureus*. During infection, osteoclasts might cause early bone resorption but may contribute to clearing bacteria and necrotic bone in later phases of infection, thus facilitating bone repair. The increased BMD observed in CCR6^−/−^ mice could likely reflect some compensatory anabolic response, potentially driven by dysregulated immune response without CCR6.

We also found a significant correlation between disease severity and the concentration of CCL20 in the sera of *S. aureus* osteomyelitis patients, particularly in patients with septic complications. Klaus et al. reported elevated plasma concentrations of CCL20 in sepsis patients, with the highest concentrations observed in those with the most severe disease (18). Nonetheless, these results suggest that CCL20 could be leveraged as a prognostic biomarker of adverse outcomes and septic complications in osteomyelitis.

**Fig 7:**
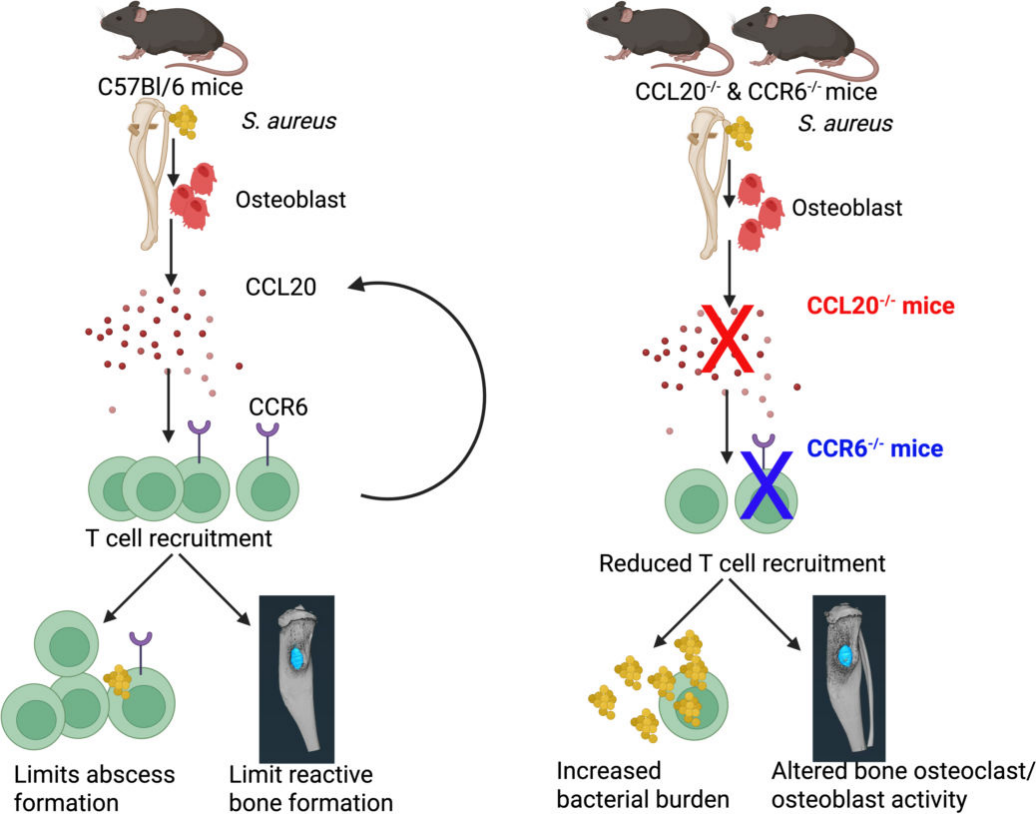
Schematic model of CCL20/CCR6-mediated immune regulation during *S. aureus* osteomyelitis. At the onset of *S. aureus* osteomyelitis, CCL20 released by osteoblasts and macrophages induces CCL20 secretion via Toll-like receptor (TLR) activation, which is crucial for the early recruitment of CCR6^+^ T cells at the site of infection to accelerate bacterial clearance and modulate disease severity in implant-associated osteomyelitis.

To summarize our findings, we propose a schematic model of how CCL20/CCR6 signaling mediates immune responses during the establishment of implant-associated *S. aureus* osteomyelitis (Fig. 7), which warrants several future investigations. First, we need to assess the complex temporal changes in the infiltrating T cells and macrophages in the bone marrow niche, which are part of CCL20/CCR6 crosstalk during *S. aureus* osteomyelitis. Second, we must investigate the molecular mechanisms behind increased reactive bone formation in CCR6^−/−^ and CCL20^−/−^ mice. Finally, to reconcile the clinical study data and observations in mice, we ought to understand the relationship between CCL20 secretion and osteomyelitis-induced sepsis in a more clinically relevant osteomyelitis model. Humanized mice, which are more susceptible to *S. aureus* osteomyelitis, could be more appropriate for such studies (54–56). Ultimately, such studies will improve our understanding of the complex host-immune mechanisms that are at play during osteomyelitis.

## MATERIALS AND METHODS

### Patient enrollment

Recruited patients were either enrolled in an international biospecimen registry (AO Trauma Clinical Priority Program [CPP] Bone Infection Registry [57]) or participated in IRB-approved clinical studies conducted at Virginia Commonwealth University (58, 59). Inclusion criteria included clinical microbiology lab confirmation of *S. aureus* implant-associated osteomyelitis and comprehensive clinical records. Exclusion criteria included concurrent inflammatory conditions, insufficient serum volume, and incomplete clinical records.

### Bacterial strains

We used a methicillin-resistant *S. aureus* (USA300 LAC) strain for *in vitro* experiments. A bioluminescent strain of USA300 LAC (USA300 LAC::lux) (54, 60, 61) was used for *in vivo* experiments.

### CCL20 *in vitro* induction assay

#### Cell isolation/culture

Primary bone marrow-derived macrophages (BMDMs), osteoblasts, and osteoclasts were differentiated using bone marrow cells from femurs and tibias of 12-week-old C57BL/6J mice (61). Briefly, after harvesting, bones were washed in R10 medium (RPMI 1640 + 10% fetal bovine serum [FBS], 1% HEPES, and 1% antimicrobial/antimycotic) before disinfection with 70% ethanol. Next, the epiphyses of the bones were cut off, and the marrow was flushed out with a 2-3G needle using PBS with 2% FBS. Bones were resuspended in R10 medium to thoroughly separate the bone marrow from the bone. Bones were then cut into small pieces and incubated in αMEM containing 10% FBS, 2 mmol·L^−1^ L-glutamine, 1% antimicrobial/antimycotic, and collagenase I (1  mg·mL^−1^, Thermo Fisher Scientific) for 90 min at 37°C to isolate osteoblasts. After this digestion process, the bone pieces were rinsed to remove unwanted marrow cells, transferred into a flask containing αMEM supplemented with 10% FBS, 2 mmol·L^−1^ L-glutamine, 1% antimicrobial/antimycotic, and incubated at 37°C in 5% CO_2_ for 3 or 4 days. To ensure cellular purity, only primary osteoblasts obtained after three to five passages were used for the CCL20 induction assays. To differentiate osteoclasts, whole bone marrow cells were cultured in R10 (RPMI+ 10% Fetal Bovine Serum) media containing macrophage colony-stimulating factor (M-CSF; 30 ng·mL^−1^, PeproTech), and RANKL (100 ng·mL^−1^, PeproTech) was then added and cultured for 5–6 days at 37°C in 5% CO_2_. BMDMs were differentiated with M-CSF (30 ng·mL^−1^, PeproTech). Murine calvarial MC3T3-E1 osteoblasts were plated in DMEM + 10% FBS, 2 mmol·L^−1^ L-glutamine, and 1% antimicrobial/antimycotic and grown to 80% confluence. RAW 264.7 cells were cultured in DMEM + 10% FBS, 2 mmol·L^−1^ L-glutamine, and 1% antimicrobial/antimycotic to 80% confluence. Subsequently, BMDMs and RAW 264.7 cells were cultured in R10 medium containing PBS, murine IFN-γ (50 ng·mL^−1^, PeproTech), or murine IL-4 (20 ng·mL^−1^, PeproTech) for 2 h to generate M0, M1, and M2 macrophages, respectively.

#### Cellular infection

Primary osteoclasts and osteoblasts, MC3T3-E1, and differentiated BMDMs and RAW 264.7 cells (M0, M1, and M2) were infected with *S. aureus* USA300 LAC at an MOI of 1, 10, and 50 for 24 h. All the *in vitro* infection studies were performed using media without antibiotics. As observed by others (62, 63), we did not see a drop in cell viability at 24 h due to infection. Cell culture supernatants were harvested after infection to measure CCL20 secretion using a Mouse CCL20 Uncoated ELISA Kit (Invitrogen). Serum CCL20 concentrations in patients were determined with a Luminex-based Milliplex xMAP Multiplex Assay (Millipore Sigma), performed according to the manufacturer’s instructions.

### *In vitro* antimicrobial assay

The MRSA was cultured in 96-well plates and treated with recombinant CCL20 (PeproTech) at 0–12.5 µg/mL concentrations. Sitafloxacin (CAS# 163253-35-8, Nanjing Chemlin Chemicals) was used as a positive control and was tested at concentrations ranging from 0 to 6.4 mg/mL. Bacterial growth in these plates was assessed as a measure of optical density (OD) at 600 nm using a spectrophotometer after 24 h of incubation at 37°C in 5% CO_2_. Antimicrobial efficacy was evaluated by calculating MIC_50_, which is defined as the concentration that achieved a 50% reduction in OD relative to the untreated control.

### *In vivo* mouse model of implant-associated *S**.** aureus*-induced osteomyelitis

The C57BL/6 mice, CCL20^−/−^ (B6[C]-Ccl20tm1b[EUCOMM]Hmgu/KmskJ; RRID:IMSR_JAX:030308), and CCR6^−/−^ (B6.129P2-Ccr6tm1Dgen/J; RRID:IMSR_JAX:005793) mice (both knockouts on the C57BL/6 background) used in the study were purchased from The Jackson Laboratory (Bar Harbor, ME, USA) and maintained in the American Association of Laboratory Care accredited animal facility at the University of Rochester. A transtibial implant-associated osteomyelitis model was used for all *in vivo S. aureus* challenge experiments in mice (54, 60, 61). Briefly, L-shaped stainless-steel implants were contaminated with USA300 LAC::lux (5.0 × 10^5^ CFU/mL) grown overnight and surgically implanted into the tibiae of mice from the medial to the lateral side. The body weight change and bioluminescence intensity at the infection site were evaluated longitudinally, and terminal assessment of CFUs (in the implant, surgical site soft tissue, and tibia) was performed at 14 days post-infection. Investigators at the University of Rochester performed all the mouse studies.

### Multiplex analysis of cytokines

Bone homogenates collected after 14 days post *S. aureus* infection were sent to Eve Technologies (Calgary, Canada) for cytokine profiling. Multiplex analysis was performed using the Luminex 200 system (Luminex, Austin, TX, USA). The mouse TH17 discovery assay was run according to the manufacturer’s protocols (MilliporeSigma, MA, USA). The panel consisted of the following cytokines: IL-17A, IL-17E, IL-17F, IL-21, IL-22, IL-23, IL-27, IL-28B, IL-31, IL-33, and TNFβ. The assay sensitivity ranged from 0.5 to 498.6 pg/mL.

### μCT imaging and analysis

Infected tibias were fixed in 10% neutral buffered formalin for 3 days at room temperature. Following fixation, samples were rinsed in PBS and distilled water and then imaged *ex vivo* by μCT in a VivaCT40 (Scanco Medical; Bassersdorf, Switzerland) with a voxel size of 10.5 µm, an energy of 55 KV, and an integration time of 300 ms at an intensity of 145 μA. The μCT DICOM scans were reconstructed using Amira software (FEI Visualization Sciences Group, Burlington, MA, USA) to generate a three-dimensional depiction of the bone tissue. An intensity threshold of >2,500 Hounsfield Units was used to designate bone as a distinct material separate from the exterior as previously described (64). The medial and lateral defect volumes were identified through manual segmentation of the void tibial cortex area and interpolation between slices. Similarly, overall tibial volume was assessed through segmentation of the tibial cortex and trabecula, while avoiding reactive bone protrusions and interpolation between slices. The remaining bone material not defined as part of the underlying tibial profile around the defects was defined as reactive bone. Medial and lateral defect volumes were calculated and normalized to total tibial volume; the volume of normalized reactive bone for each tibia was also measured.

### Histology

Histopathological analyses were performed according to protocols described previously (54, 60, 61). Briefly, after the μCT measurement, each mouse tibia was decalcified with 14% EDTA disodium salt dihydrate (pH 7.4) for 2 weeks at room temperature. All samples were embedded in paraffin and sectioned at a thickness of 5  µm. After staining, digital images of the serially stained slides were acquired using a VS120 Virtual Slide Microscope (Olympus, Waltham, MA, USA). To compare the number of osteoclasts within the infected tibia, TRAP staining was performed. The intensity of TRAP staining within each ROI in the infection site, cortical bone, and trabecular bone regions in each experimental group was quantified using colorimetric histomorphometry with a custom Analysis Protocol Package in Visiopharm (v.2019.07; Hoersholm, Denmark). Tibial slides were also stained with Brown-Brenn, a modified Gram stain to visualize gram-positive bacteria. This results in gram-positive organisms stained dark purple, cell nuclei stained pink, and connective tissue stained yellow. Slides were digitized using a VS110/VS120 Virtual Slide Microscope (Olympus, Waltham, MA). SAC area was calculated and normalized to the total bone area averaged across three histological levels, each with 4–6 biological replicates, using Olympus OlyVIA software.

### Multicolor immunofluorescence staining

#### Primary antibodies

The following antibodies were utilized for immunostaining: goat anti-CD3-epsilon (clone M-20, sc-1127, RRID: AB_631128, Santa Cruz Biotechnology) at a 1:100 dilution, rabbit anti-CCL20 (Cat no. BS-1268R, Bioss Antibodies), rabbit-anti-CCR6 (Cat no. orb253812, Biorbyt), rat anti-mouse F4/80 (clone Cl:A3-1, MCA497G, RRID:AB_872005, BioRad), biotin rat anti-Ly6G (clone 1A8, 127604, RRID: AB_1186108, Biolegend), and hamster anti-mouse CD11c (MBS690274, MyBiosource). All other primary antibodies were used at a 1:50 dilution.

#### Secondary antibodies

The following antibodies were utilized for immunostaining: Alexa Fluor 568-conjugated donkey anti-goat IgG (A-11057, RRID: AB_2534104, Thermo Fisher Scientific) at a 1:200 dilution to detect CD3-epsilon, Alexa Fluor 488-conjugated donkey anti-rabbit IgG (711-546-152, RRID: AB_2340619, Jackson ImmunoResearch Laboratories) at a 1:200 dilution for CCL20 and CCR6 detection, FITC-conjugated anti-Syrian hamster IgG (307-096-003, RRID: AB_2339583, Jackson ImmunoResearch Laboratories) at a 1:200 dilution for CD11c, Alexa Fluor 647 donkey anti-rat IgG (712-606-153, RRID: AB_2340696, Jackson ImmunoResearch Laboratories), and Alexa Fluor 680-conjugated streptavidin (S32358, Thermo Fisher Scientific) at a 1:200 dilution to detect F4/80 and Ly6G, respectively.

#### Staining protocol

Briefly, 5 µm formalin-fixed paraffin sections were incubated at 60°C overnight for deparaffinization. The tissue sections were quickly transferred to xylene and gradually hydrated by sequential transfer to absolute alcohol, 96% alcohol, 70% alcohol, and finally water. Subsequently, the sections were immersed in Antigen Unmasking Solution (Vector Laboratories) and boiled for 2 h. Nonspecific binding was blocked with 5% normal donkey serum in Tris-buffered saline (TBS) containing 0.5% Triton X-100 for 40 min at room tempertature in a humidified chamber. Primary antibodies at appropriate concentrations were then added to these sections and incubated at 4°C overnight. This was followed by washing with PBS and incubation with a secondary antibody at RT for 2 h. Finally, the slides were rinsed for 1 h in PBS and mounted with Vectashield antifade mounting medium with DAPI (OM-1200, Vector Laboratories, Burlingame, CA, USA). Images were acquired with a Zeiss Axioplan 2 microscope connected to a Hamamatsu camera. The images were analyzed using ImageJ, a Java-based image processing program, developed at the National Institutes of Health (Bethesda, MD, USA), version 1.53. The ratio of the raw integrated density of each marker was measured and normalized with the DAPI. DAPI intensity per field was further calculated to avoid the potential bias due to defective recruitment as an additional normalization method.

### Single-cell suspensions and flow cytometry

Following euthanasia, BMCs were isolated by flushing the bone with 1 mL of PBS + 2% FBS. Immunophenotyping of the bone marrow from sterile and infected C57BL/6, CCR6^−/−^, and CCL20^−/−^ mice was performed. Briefly, 10^6^ cells/mouse were initially stained with fixable viability dye eFluor 520 (eBioscience, Thermo Fisher Scientific catalog: 65-0867-14) for 30 min at 4°C to exclude dead cells from the analysis. Following washing, surface antibody cocktails were added for 80 min at 4°C (anti-CD45 APC-Cy7 (Biolegend catalog 103116); anti-CD3 BV41 (Biolegend catalog 100227); anti-CD4 BV510 (BD Biosciences catalog 569249); anti-CD8 BUV395 (BD Biosciences catalog 565968); anti-F480 PerCP (Biolegend catalog 123125); anti-CD86 BV650 (BD Biosciences catalog 564200); anti-CD163 BUV615 (Thermo Fisher Scientific catalog 366-1631-82); anti-CCR6 APC (Biolegend catalog 129813). After staining, the cells were fixed with 2% formaldehyde/water before running on a Cytek Aurora five-laser spectral flow cytometer (Cytek Biosciences). Flow data were analyzed using the OMIQ software from Dotmatics (https://www.omiq.ai/). Single-color compensation controls for these antibodies were created using UltraComp eBeads Plus Compensation beads (Thermo Fisher Scientific, catalog 01-3333-43). All antibodies were purchased from BioLegend, BD Biosciences (San Jose, CA, USA), or Thermo Fisher Scientific.

### DEXA scan

BMD was assessed in all mice using a DEXA scanner (iNSiGHT VET DXA; OsteoSys, Seoul, Korea). Mice were anesthetized during imaging via vaporized isoflurane, and each mouse was placed on the scanner bed within the designated scanning area (16.5 cm × 25.5 cm) in the prone position with the lower limbs stretched away from the abdomen. The Insight DEXA employed a scan time of 30 seconds and utilized a cone beam scanning method, generating beams with 60 and 80 kV and 0.8 mA that provided up to a 100 micron resolution for each image. The system provides quantitative data on the bone tissue, fat tissue content, lean tissue content, and the total tissue mass within the region of interest (ROI). Exclusion ROI was used to highlight the skull, ear tag, and nose cone to remove interference as recommended by the manufacturer. The iNSiGHT VET DXA was calibrated daily before testing using a quality control phantom according to the manufacturer’s instructions. To calculate BMD, an ROI was drawn defining the region of the tibia. The DEXA scan was done both on day 14 post-infection and compared to the scan before surgery.

### Statistics

For statistical analyses involving more than two groups, we utilized the nonparametric Kruskal‒Wallis test, one-way ANOVA, and two-way repeated measures ANOVA. Unpaired Student’s *t*-tests were used to assess the significance of differences between the two experimental groups. The data are presented as the means ± SEM. A *P*-value of <0.05 was considered significant.

## SUPPLEMENTAL MATERIAL

Figure S1 mbio.01413-25-s0001.tiff Systemic CCL20 secretion in mice due to *S. aureus* osteomyelitis.

Figure S2 mbio.01413-25-s0002.tiff Production of CCL20 is diminished in CCL20^−/−^ mice.

Figure S3 mbio.01413-25-s0003.tiff Temporal changes in body weight during osteomyelitis to estimate *S. aureus*-driven morbidity.

Figure S4 mbio.01413-25-s0004.tif Quantification of DAPI-stained area per field across C57BL/6, CCR6^−/−^, and CCL20^−/−^ mice.

Figure S5 mbio.01413-25-s0005.tif CCL20/CCR6 essential for recruitment of macrophages to the site of infection.

Figure S6 mbio.01413-25-s0006.tif Flow cytometric analysis of macrophage and CCR6⁺ macrophage recruitment at the site of infection during implant-associated *S. aureus* osteomyelitis.

Figure S7 mbio.01413-25-s0007.tiff BMD changes following *S. aureus* infection in C57BL/6, CCR6^−/−^, and CCL20^−/−^ mice.

Figure S8 mbio.01413-25-s0008.tif Comparative antibacterial effects of CCL20 and sitafloxacin.

Legends mbio.01413-25-s0009.docx Legends for Figures S1 to S8.

ASM does not own the copyrights to Supplemental Material that may be linked to, or accessed through, an article. The authors have granted ASM a non-exclusive, world-wide license to publish the Supplemental Material files. Please contact the corresponding author directly for reuse.
