# Supplementary figures and images for: CCL20/CCR6 signaling modulates disease severity during the establishment of Staphylococcus aureus osteomyelitis
Source: mBio. 2025 Aug 25;16(10):e01413-25. doi: 10.1128/mbio.01413-25 (PMC12506002; doi:10.1128/mbio.01413-25)

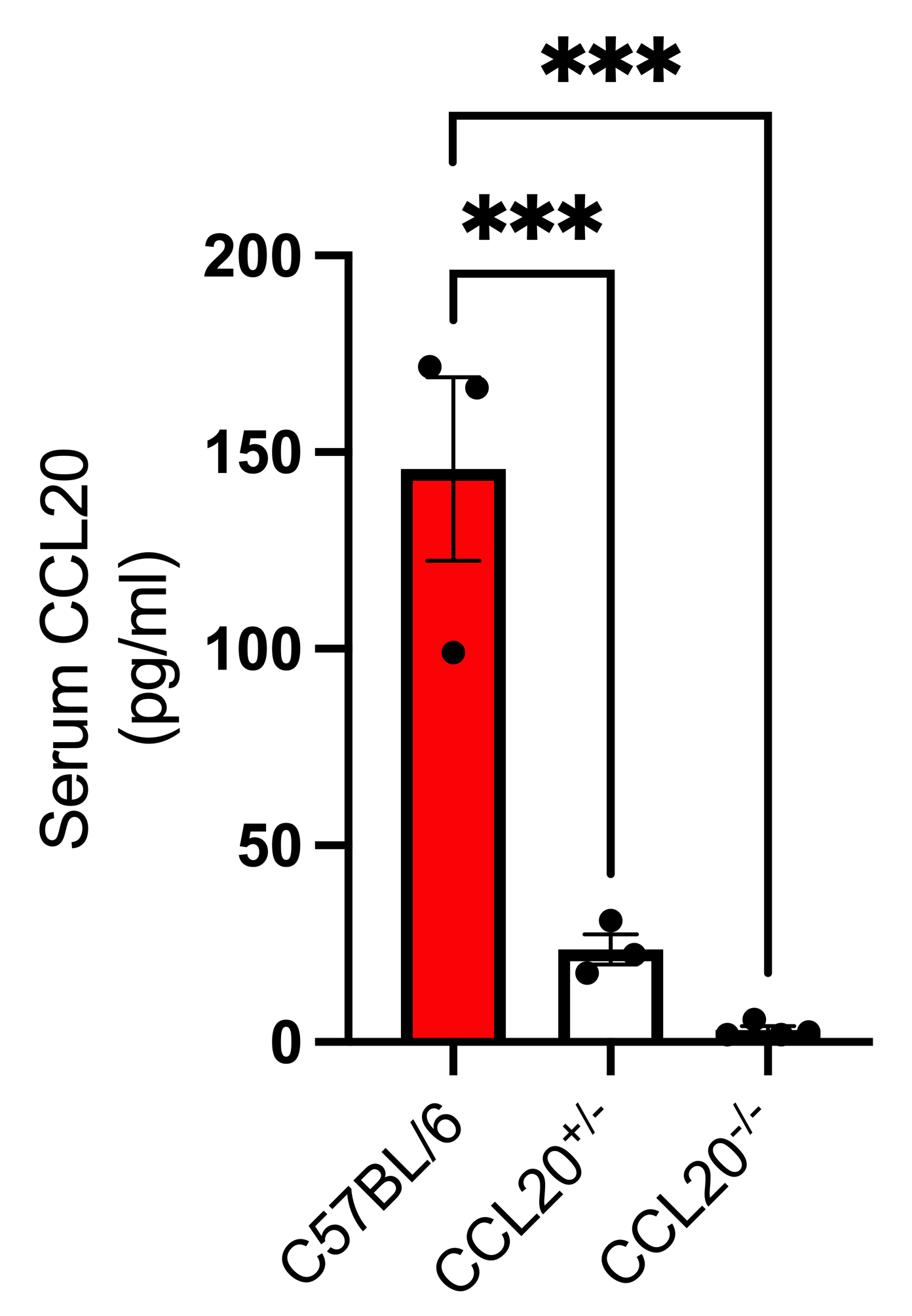

Supplement: Figure S1 — Systemic CCL20 secretion in mice due to S. aureus osteomyelitis. [file mbio.01413-25-s0001.tiff]

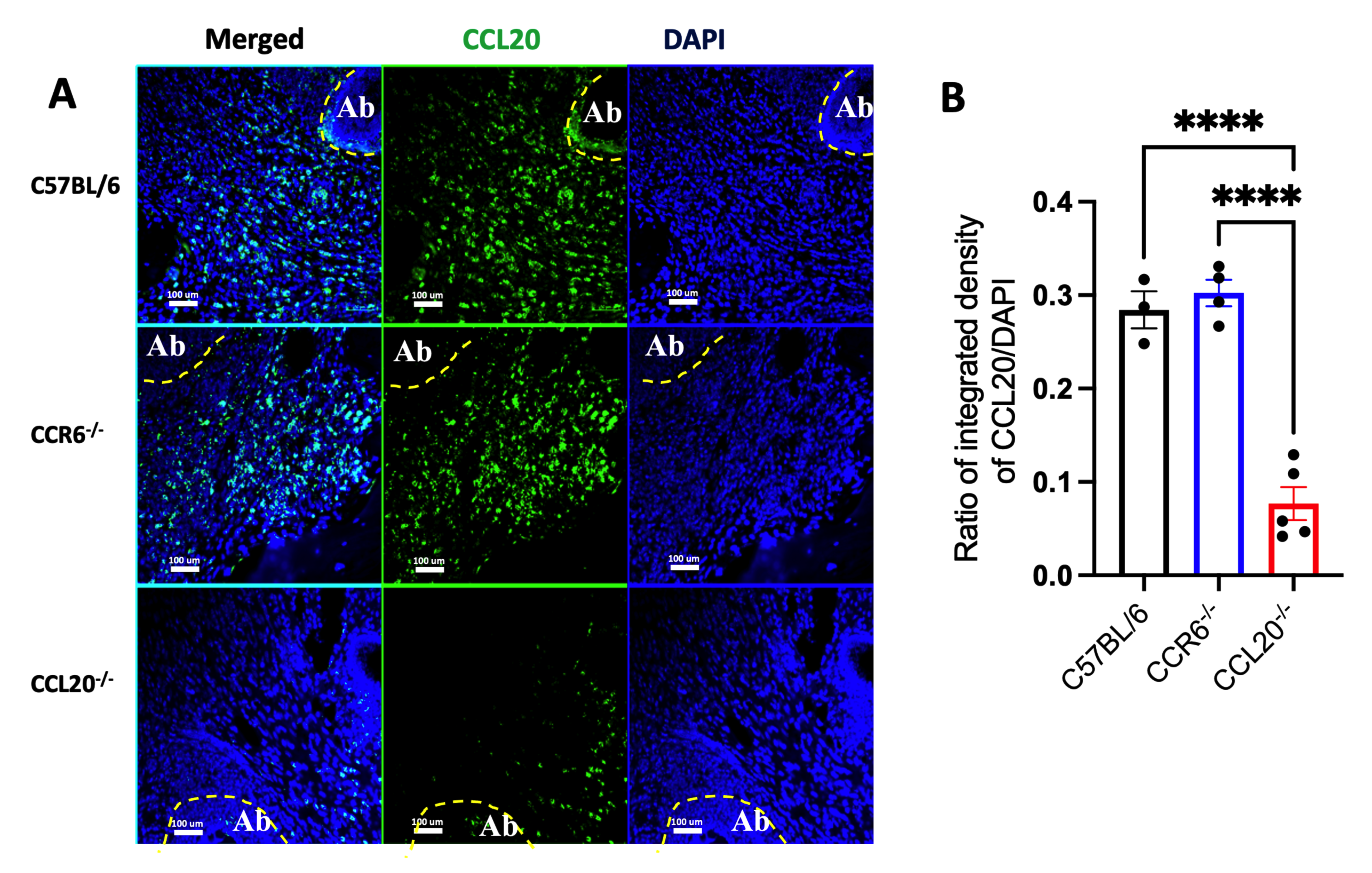

Supplement: Figure S2 — Production of CCL20 is diminished in CCL20−/− mice. [file mbio.01413-25-s0002.tiff]

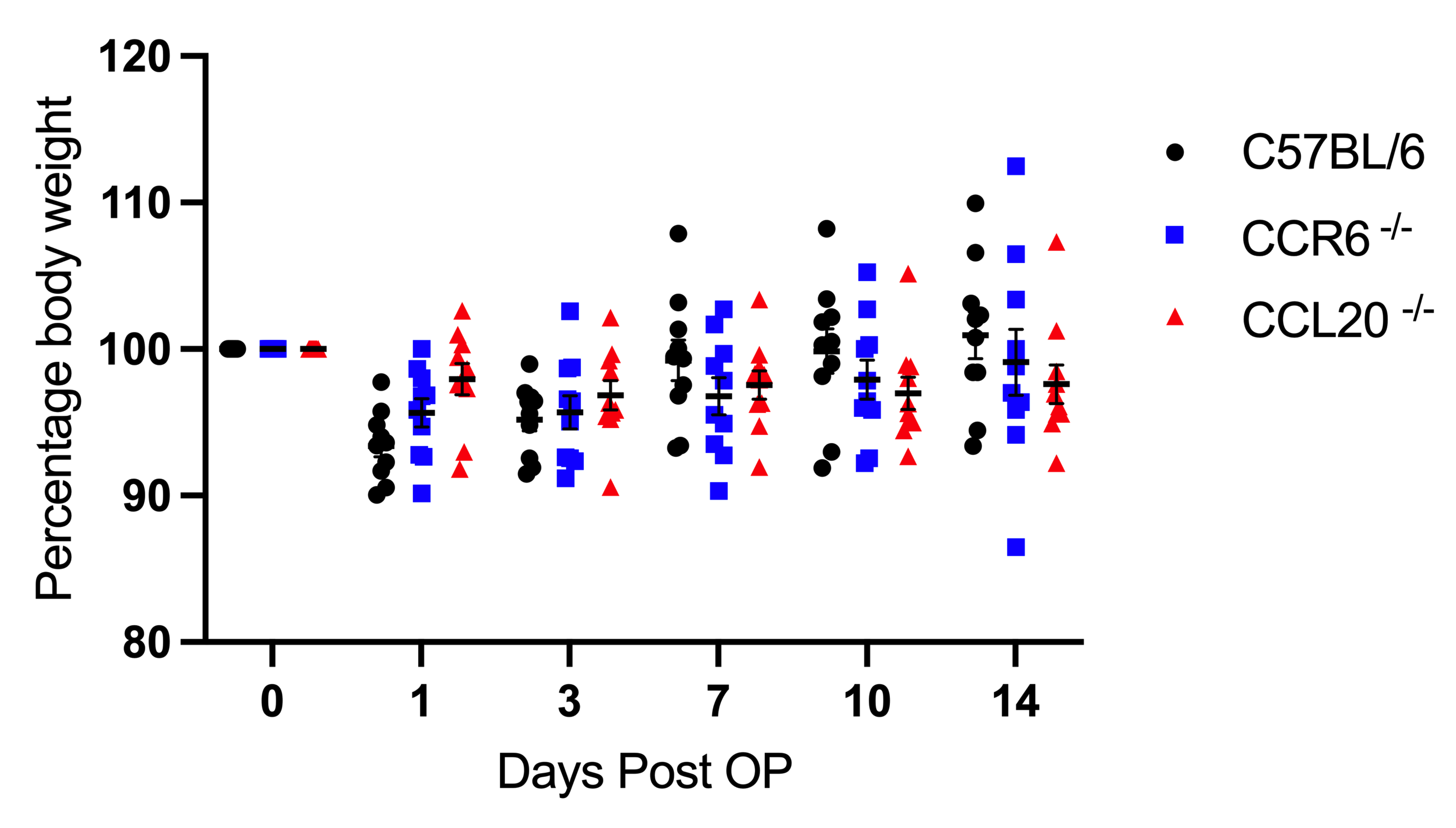

Supplement: Figure S3 — Temporal changes in body weight during osteomyelitis to estimate S. aureus-driven morbidity. [file mbio.01413-25-s0003.tiff]

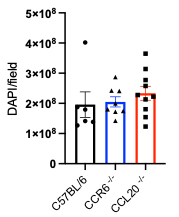

Supplement: Figure S4 — Quantification of DAPI-stained area per field across C57BL/6, CCR6−/−, and CCL20−/− mice. [file mbio.01413-25-s0004.tif]

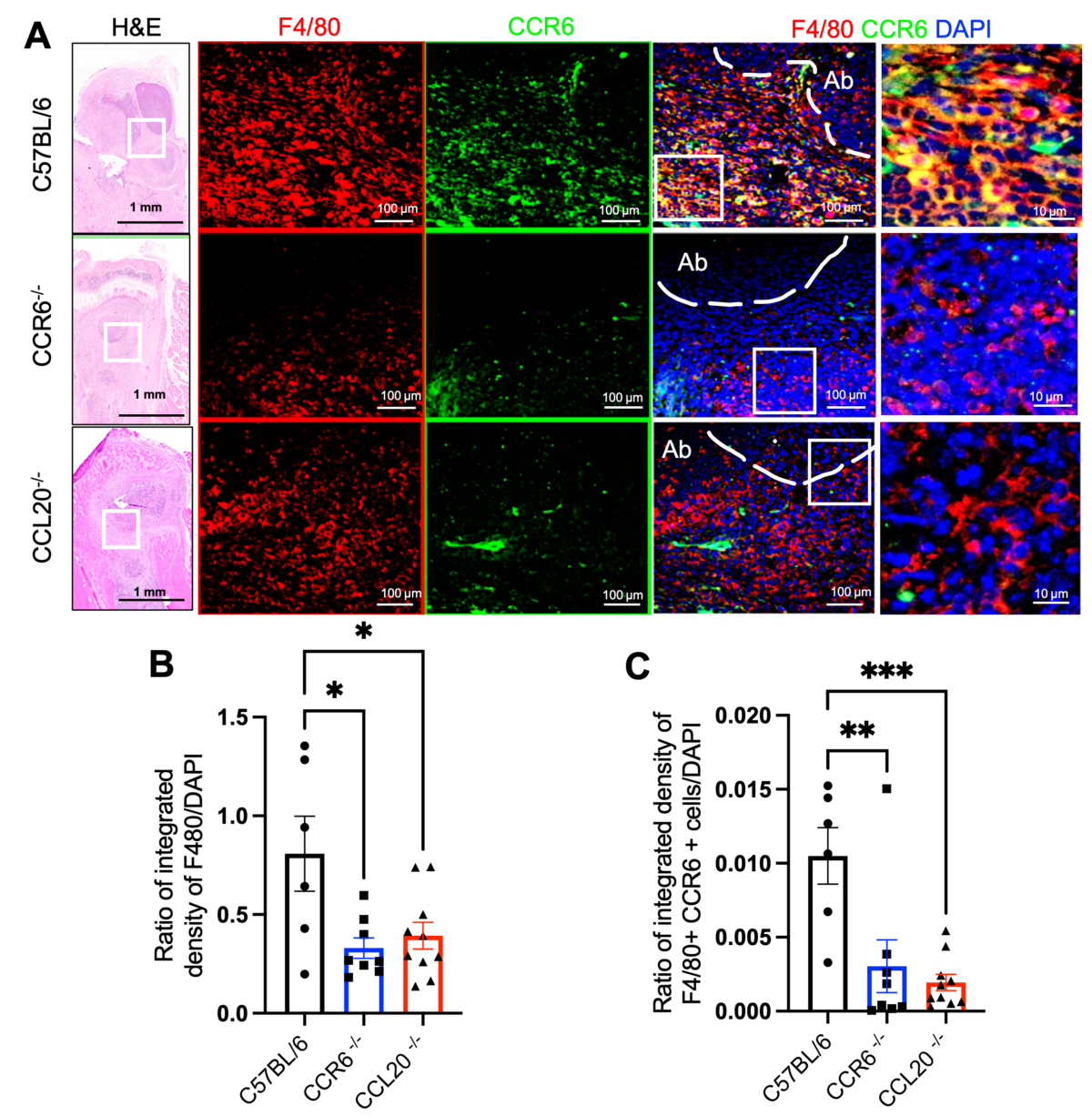

Supplement: Figure S5 — CCL20/CCR6 essential for recruitment of macrophages to the site of infection. [file mbio.01413-25-s0005.tif]

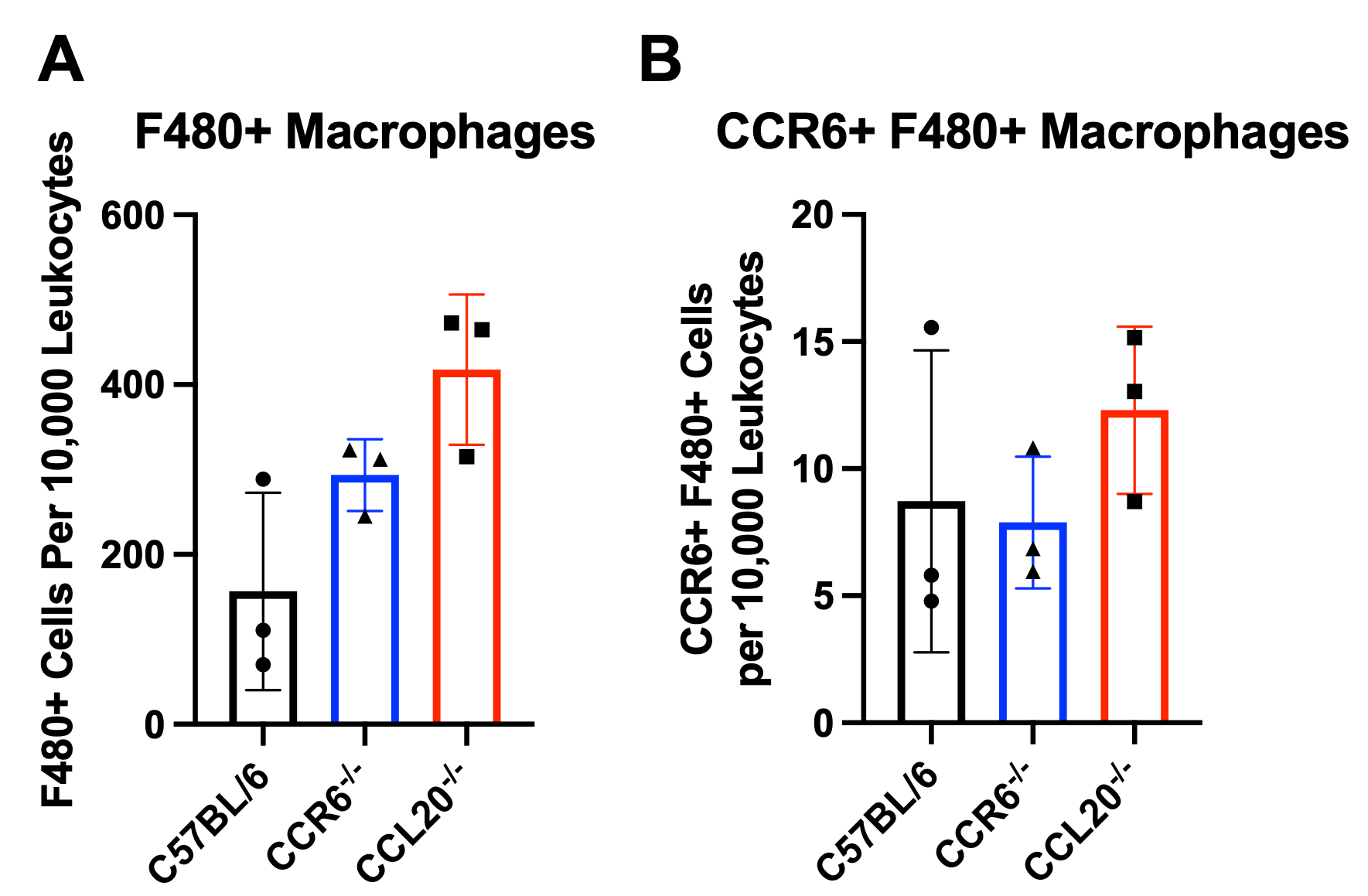

Supplement: Figure S6 — Flow cytometric analysis of macrophage and CCR6⁺ macrophage recruitment at the site of infection during implant-associated S. aureus osteomyelitis. [file mbio.01413-25-s0006.tif]

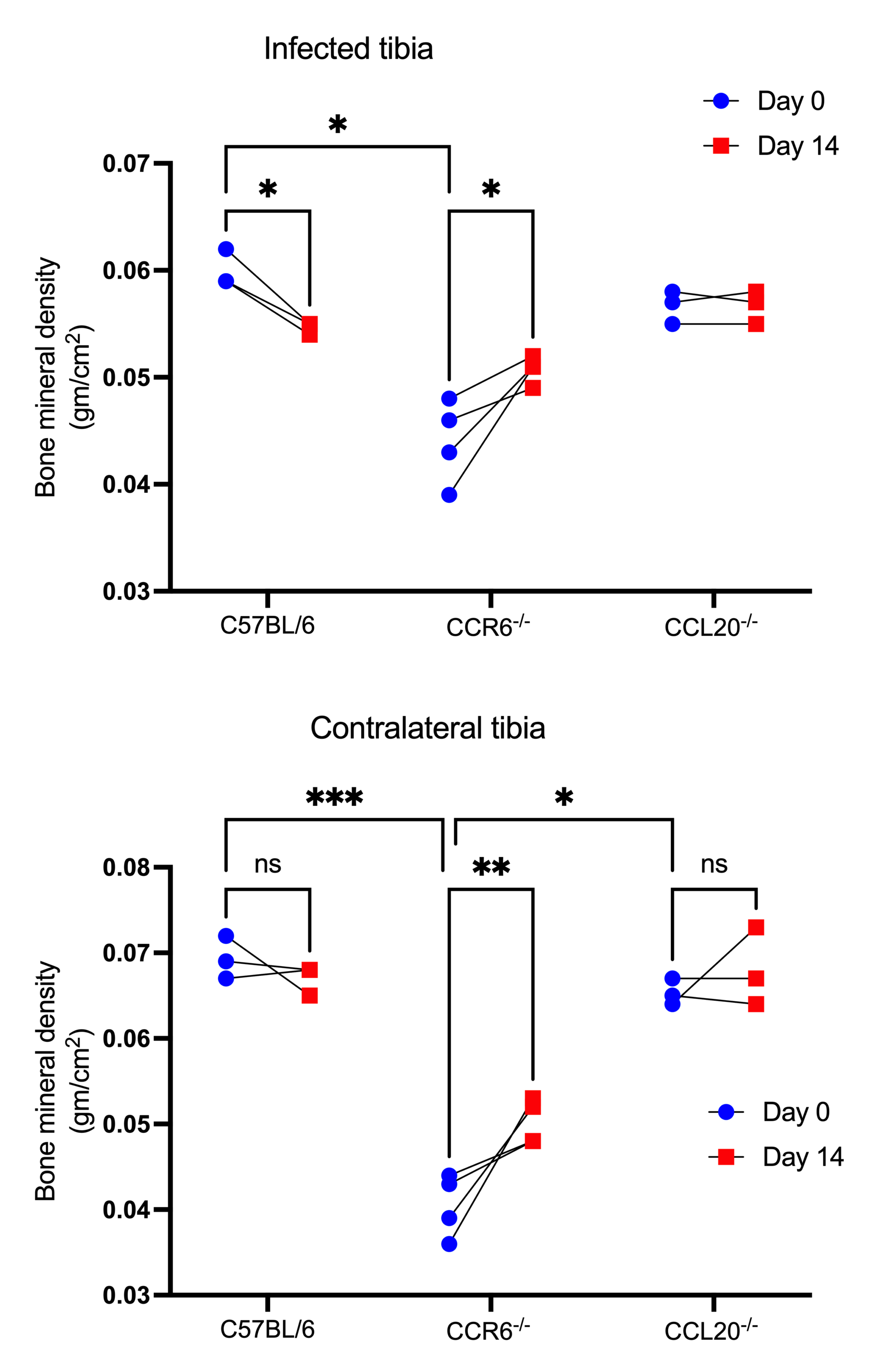

Supplement: Figure S7 — BMD changes following S. aureus infection in C57BL/6, CCR6−/−, and CCL20−/− mice. [file mbio.01413-25-s0007.tiff]

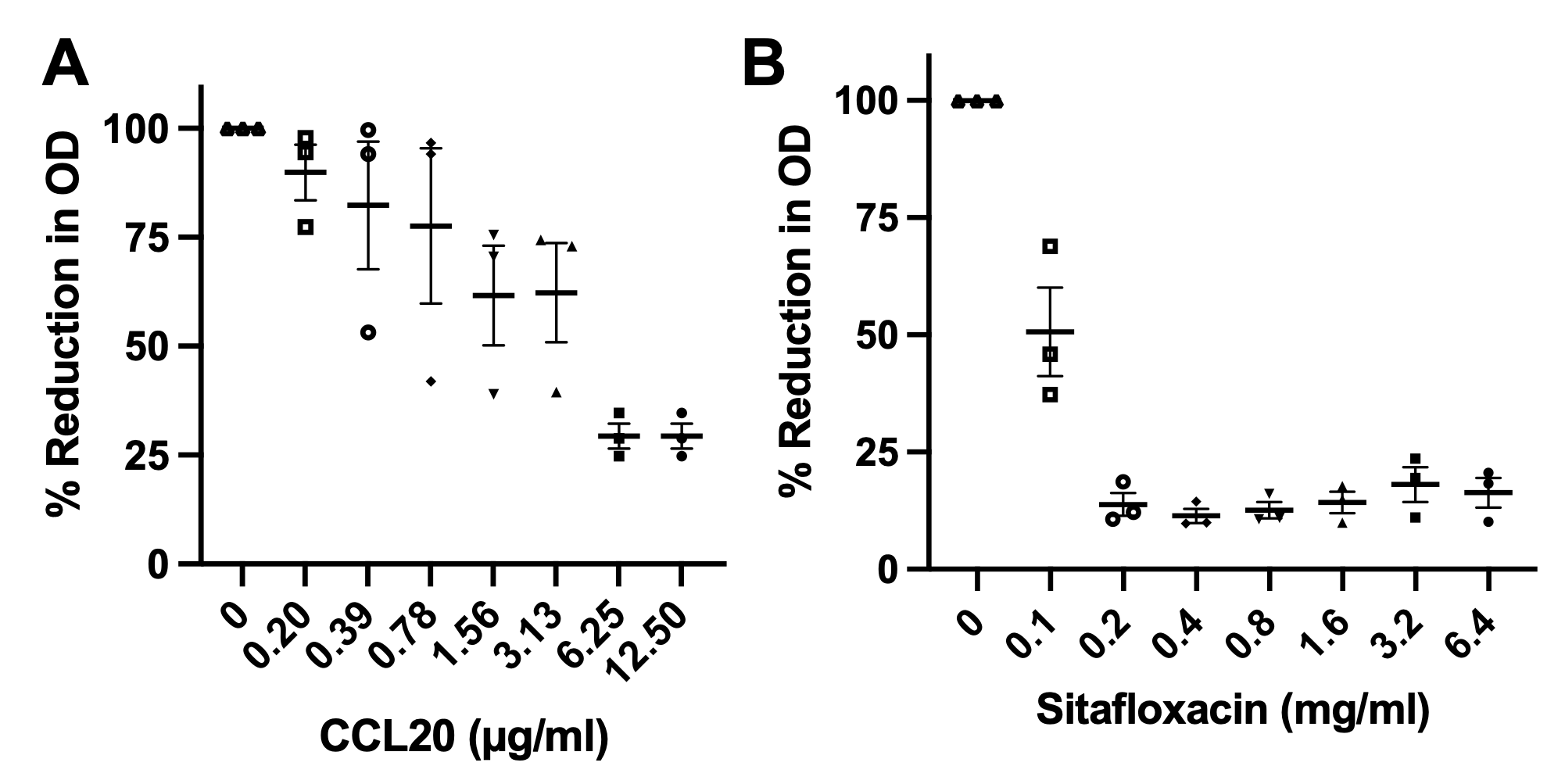

Supplement: Figure S8 — Comparative antibacterial effects of CCL20 and sitafloxacin. [file mbio.01413-25-s0008.tif]
