# Supplementary material for: CCL20/CCR6 signaling modulates disease severity during the establishment of Staphylococcus aureus osteomyelitis
Source: mBio. 2025 Aug 25;16(10):e01413-25. doi: 10.1128/mbio.01413-25 (PMC12506002; doi:10.1128/mbio.01413-25)
Supplement: Legends — for Figures S1 to S8. [file mbio.01413-25-s0009.docx]

**CCL20/CCR6 signaling modulates disease severity during the establishment of *Staphylococcus aureus* osteomyelitis**

Himanshu Meghwani^a^, Javier Rangel-Moreno^b^, Kyra M. Sandercock^a^, Motoo Saito^a^,

Katya A. McDonald^a,^ Chloe M. Kraft^a^, Robert Constantine^a^, Sophia Lenigk^a^, Adryiana Rodriguez^a^, Stephen L. Kates^c^, Jennifer H. Jonason^a,d^, Edward M. Schwarz^a,d^

and Gowrishankar Muthukrishnan^a,d#^.

^a^ Center for Musculoskeletal Research, Department of Orthopaedics, University of Rochester Medical Center, Rochester, NY, USA,
^b^ Division of Allergy, Immunology and Rheumatology, Department of Medicine, University of Rochester Medical Center, Rochester, NY, USA.
^c^ Department of Orthopaedics, Virginia Commonwealth University, Richmond, VA, USA

^d^ Department of Microbiology and Immunology, University of Rochester Medical Center, Rochester, NY, USA

**Supplemental Figure Legends**

**Supplemental Figure 1. Systemic CCL20 secretion in mice due to *S. aureus* osteomyelitis.** C57BL/6, CCL20 heterozygous, and CCL20 homozygous knockout mice were sacrificed on day 14 post S. aureus infection. CCL20 was measured in the sera by ELISA, and the data are presented as mean  ±  SEM (pg/mL, n = 3-4 mice/experimental group, **p < 0.01, one-way ANOVA)

**Supplemental Figure 2. Production of CCL20 is diminished in CCL20^-/-^ mice.** A) *S. aureus stimulat*es CCL20 production in the bone surrounding the infection site. 5 µm tibial sections from C57BL/6, CCR6-/, and CCL20^-/-^ mice, 14 days post-surgery, were stained with anti-CCL20 antibody and visualized using a fluorescence microscope. **A**) The images of tibial sections adjacent to the abscess area (Ab), stained for CCL20 (green) and nucleus (DAPI, blue), are displayed here (scale bar = 100 µm). **B**) Histomorphometric analysis was conducted by measuring the ratio of the raw integrated density of CCL20/DAPI. The data are presented as Mean ± SEM for the experimental groups (n = 3-4, ****p < 0.0001, one-way ANOVA).

**Supplemental Figure 3. Temporal changes in body weight during osteomyelitis to estimate *S. aureus*-driven morbidity**. Mice were weighed at day 0 and then at several days post-infection to calculate changes in body weight over time after *S. aureus* infection (n= 9-12).

**Supplemental Figure 4. Quantification of DAPI-stained area per field across C57BL/6, CCR6-/-, and CCL20-/- mice.** DAPI staining was utilized to assess cellular density at the site of infection across genotypes post-infection. The DAPI-positive area per field was consistent across all three genotypes, indicating that differences observed in other parameters are not attributable to variations in overall cellular density. Data represent mean ± SEM (n = 3–4 mice per group, with 2–3 tissue levels analyzed per tibia).

**Supplemental Figure 5. CCL20/CCR6 essential for recruitment of macrophages to the site of infection.** Tibiae sections from C57BL/6, CCR6^-/-^ and CCL20^-/-^ mice 14 days post-infection were processed for immunofluorescent microscopy. **A)** Representative H&E images of tibial sections containing abscesses (Ab). Serial sections were stained with antibodies to detect macrophages (F4/80, red), CCR6 (green). Nuclei were labeled with DAPI. Scale bar = 100 µm. **B)** Histomorphometric analysis depicts the ratio of raw integrated density between **C)** CCR6/DAPI, C) F4/80/DAPI, and **D)** CCR6+ F4/80+ macrophages/DAPI. The data represent the Mean ±  SEM for the groups (n =  3-4 mice/group). One-way ANOVA was used to calculate statistical significance (*p < 0.05, **p < 0.01, ***p < 0.001, ****p < 0.0001).

**Supplemental Figure 6. Flow cytometric analysis of macrophage and CCR6⁺ macrophage recruitment at the site of infection during implant-associated *S. aureus* osteomyelitis.** A multicolor spectral flow cytometry panel was developed, optimized, and applied to analyze tibial bone marrow cells (BMCs) from C57BL/6, CCL20⁻/⁻, and CCR6⁻/⁻ mice. **(A)** Total macrophages (CD45⁺/F4/80⁺) and **(B)** CCR6⁺ macrophages (CCR6⁺/F4/80⁺) were quantified. No significant differences in macrophage recruitment were observed in CCL20⁻/⁻ or CCR6⁻/⁻ mice compared to wild-type controls. Data are presented as mean ± SEM (n = 3 mice per group).

**Supplemental Figure 7. Bone mineral density (BMD) changes following *S. aureus* infection in C57BL/6, CCR6^-/-^, and CCL20^-/-^ mice.** BMD was measured using dual-energy X-ray absorptiometry (DEXA) at day 0 and day 14 post-infection in both the infected and contralateral tibiae (n = 3–4 mice per group). Results are reported in g/cm³ and presented as mean ± SEM. Statistical significance was determined using one-way ANOVA (* p < 0.05; ** p < 0.01; *** p < 0.001).

**Supplemental Figure 8. Comparative antibacterial effects of CCL20 and Sitafloxacin.** Optical density (OD) measurements after 24 hours of treatment demonstrate the antibacterial activity of (A) CCL20 and (B) Sitafloxacin. Data are presented as mean ± SEM (n = 3).
